# Supplementary material for: Application of a concise video to improve patient understanding of tumor genomic testing in community and academic practice settings
Source: PLoS One. 2026 Oct 13;21(10):e0360611. doi: 10.1371/journal.pone.0360611 (PMC13660886; doi:10.1371/journal.pone.0360611)
Supplement: Supplementary file 1 [file pone.0360611.s001.pdf]

Supplementary Figure S1. Study Overview, Accrual, and Metrics. (**A**) Patient flow diagram for study. (**B**) Number of participants accrued per month (y-axis) by time in month (x-axis). Accrual cohort indicated by color – Cohort 1: Breast (pink), Cohort 2: Lung (purple); Cohort 3: Other (blue); Cohort 4: Community (green). (**C**) Study instrument utilization by time point.

A

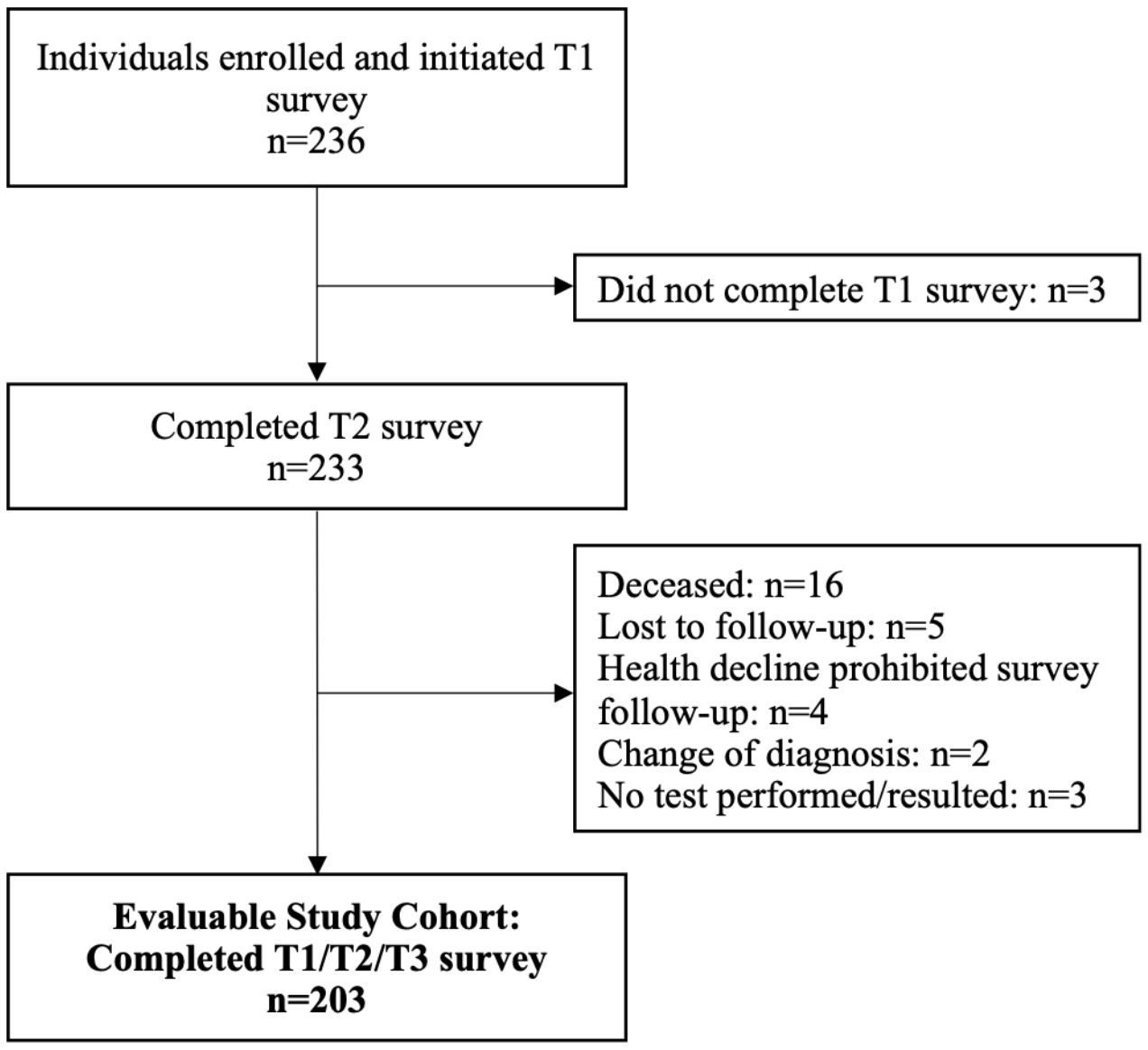

B

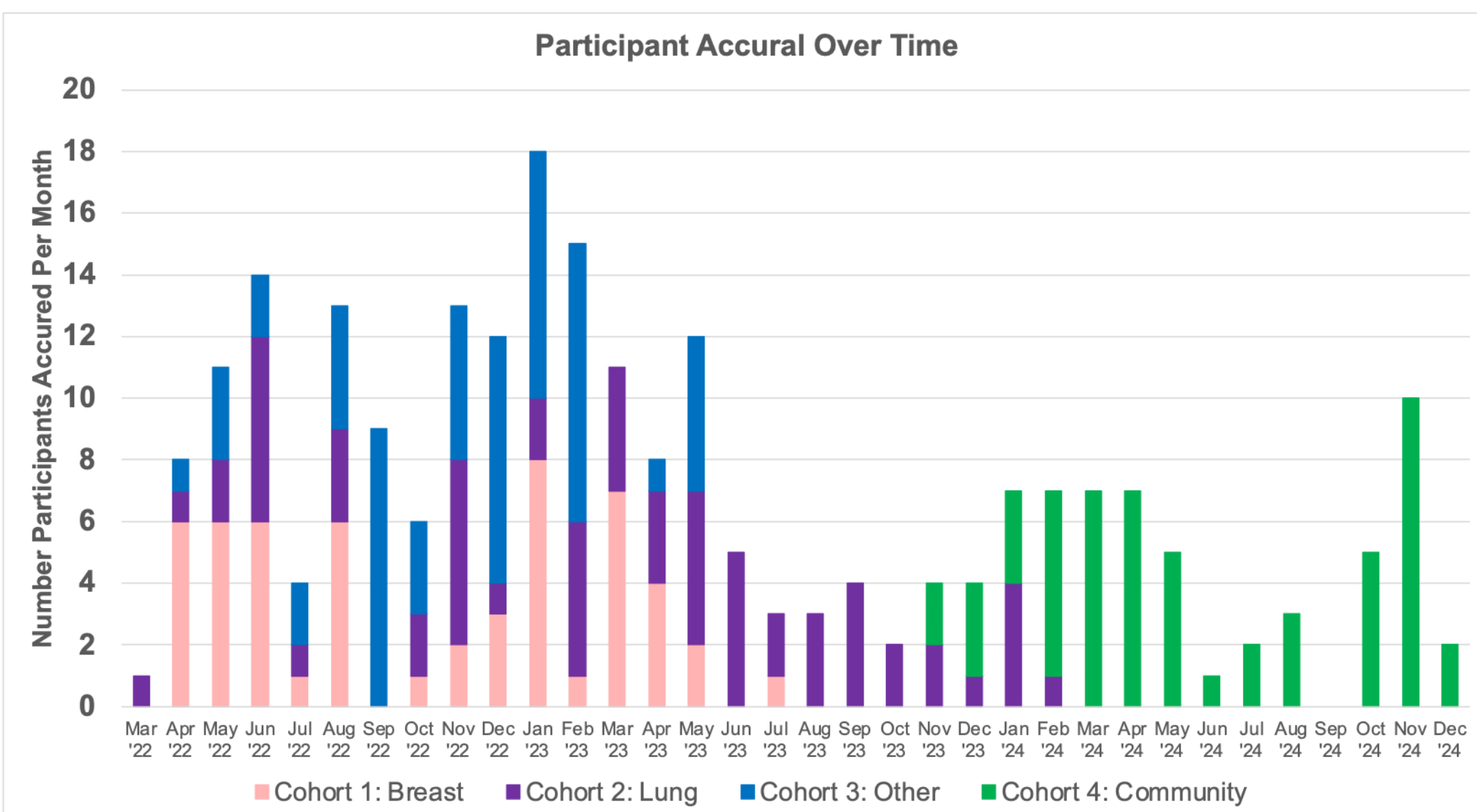

C

Instrument	T1<br>Before Video-Viewing	T2<br>After Video-Viewing	T3<br>After return of participant's results
Demographics	✓		
General Genomic Knowledge/Understanding (GKU)	✓	✓	✓
Video-message Specific Knowledge (VMSK)	✓	✓	✓
Attitudes Around Tumor Genomic Testing (ATG)	✓	✓	✓
Trust in Physician/Provider (TIP)	✓	✓	✓
Tumor Genomic Testing Intentions	✓	✓	
Video-Specific Opinions		✓	
