# Supplementary material for: Application of a concise video to improve patient understanding of tumor genomic testing in community and academic practice settings
Source: PLoS One. 2026 Oct 13;21(10):e0360611. doi: 10.1371/journal.pone.0360611 (PMC13660886; doi:10.1371/journal.pone.0360611)
Supplement: Supplementary file 2 [file pone.0360611.s002.pdf]

Supplementary Table S1. Cohort 1-Breast Cancer correct responses to video message-specific questions by timepoint

True/False Statement	Participants correct at T1		Participants correct at T2		Participants correct at T3		Change T1:T3	
	#	%	#	%	#	%	#	p-value
We have genes in every cell of our bodies.	46	92.0	50	100	48	96.0	+2	0.317
Tumor genomic testing might help your doctor make decisions about your cancer treatment.	49	98.0	50	100	50	100	+1	NA
Tumor genomic testing always determines what treatment a person will have.	41	82.0	49	98.0	48	96.0	+2	0.317
I must have tumor genomic testing to continue with cancer treatment.	48	96.0	48	96.0	48	96.0	0	1
My doctor has other tools besides tumor genomic testing to use to choose treatments for me.	48	96.0	49	98.0	49	98.0	+1	0.564
Tumor tissue genomic results sometimes raise more questions that require more genetic testing.	41	82.0	49	98.0	45	90.0	+4	0.248
The information that I get from tumor tissue genomic testing could be valuable to my children and other family members.	49	98.0	50	100	50	100	+1	NA
When my doctor has my results, they might recommend me to see a genetics specialist.	44	88.0	49	98.0	46	92.0	+2	0.480
The expense of tumor genomic testing is not typically covered by health insurance.	30	60.0	42	84.0	32	64.0	+2	0.593
If you do not have health insurance, you cannot have tumor genomic tissue testing performed.	48	96.0	48	96.0	48	96.0	0	1
