# Supplementary material for: Application of a concise video to improve patient understanding of tumor genomic testing in community and academic practice settings
Source: PLoS One. 2026 Oct 13;21(10):e0360611. doi: 10.1371/journal.pone.0360611 (PMC13660886; doi:10.1371/journal.pone.0360611)
Supplement: Supplementary file 3 [file pone.0360611.s003.pdf]

Supplementary Table S2. Cohort 2-Lung Cancer correct responses to video message-specific questions by timepoint

True/False Statement	Participants correct at T1		Participants correct at T2		Participants correct at T3		Change T1:T3	
	#	%	#	%	#	%	#	p-value
We have genes in every cell of our bodies.	48	94.1	51	100	47	92.2	-1	0.655
Tumor genomic testing might help your doctor make decisions about your cancer treatment.	51	100	51	100	51	100	0	NA
Tumor genomic testing always determines what treatment a person will have.	37	72.6	44	86.3	37	72.6	0	NA
I must have tumor genomic testing to continue with cancer treatment.	44	86.3	48	94.1	46	90.2	+2	0.480
My doctor has other tools besides tumor genomic testing to use to choose treatments for me.	49	96.1	50	98.0	51	100	+2	NA
Tumor tissue genomic results sometimes raise more questions that require more genetic testing.	44	86.3	48	94.1	47	92.2	+3	0.257
The information that I get from tumor tissue genomic testing could be valuable to my children and other family members.	49	96.1	50	98.0	49	96.1	0	1
When my doctor has my results, they might recommend me to see a genetics specialist.	44	86.3	49	96.1	46	90.2	+2	0.480
The expense of tumor genomic testing is not typically covered by health insurance.	29	56.9	39	76.5	35	68.6	+6	0.180
If you do not have health insurance, you cannot have tumor genomic tissue testing performed.	46	90.2	47	92.2	48	94.1	+2	0.480
