# Supplementary material for: Application of a concise video to improve patient understanding of tumor genomic testing in community and academic practice settings
Source: PLoS One. 2026 Oct 13;21(10):e0360611. doi: 10.1371/journal.pone.0360611 (PMC13660886; doi:10.1371/journal.pone.0360611)
Supplement: Supplementary file 5 [file pone.0360611.s005.pdf]

Supplementary Table S4. Cohort 3-Community correct responses to video message-specific questions by timepoint. Significant results ($p<0.05$) indicated in bold.

True/False Statement	Participants correct at T1		Participants correct at T2		Participants correct at T3		Change T1:T3	
	#	%	#	%	#	%	#	p-value
We have genes in every cell of our bodies.	52	100	52	100	50	96.2	-2	NA
Tumor genomic testing might help your doctor make decisions about your cancer treatment.	50	96.2	51	98.1	49	94.2	-1	0.564
Tumor genomic testing always determines what treatment a person will have.	37	71.2	39	75.0	42	80.8	+5	0.166
I must have tumor genomic testing to continue with cancer treatment.	42	80.8	43	82.7	48	92.3	+6	0.058
My doctor has other tools besides tumor genomic testing to use to choose treatments for me.	48	92.3	52	100	49	94.2	+1	0.706
Tumor tissue genomic results sometimes raise more questions that require more genetic testing.	48	92.3	48	92.3	49	94.2	+1	0.706
The information that I get from tumor tissue genomic testing could be valuable to my children and other family members.	51	98.1	52	100	51	98.1	0	NA
When my doctor has my results, they might recommend me to see a genetics specialist.	45	86.5	50	100	48	92.3	+3	0.317
The expense of tumor genomic testing is not typically covered by health insurance.	28	53.9	40	76.9	42	80.8	+14	**0.006**
If you do not have health insurance, you cannot have tumor genomic tissue testing performed.	43	82.7	50	96.2	46	88.5	+5	0.166
