# Supplementary material for: Application of a concise video to improve patient understanding of tumor genomic testing in community and academic practice settings
Source: PLoS One. 2026 Oct 13;21(10):e0360611. doi: 10.1371/journal.pone.0360611 (PMC13660886; doi:10.1371/journal.pone.0360611)
Supplement: Supplementary file 6 [file pone.0360611.s007.pdf]

Application of a Concise Video to Improve Patient Understanding of Tumor Genomic Testing in Community and Academic Practice Settings

Deloris J. Veney MACPR[1], Lai Wei, PhD, MS[2]; Jaden Miller[3]; Amanda E. Toland, Ph D[3,4]; Carolyn J. Presley, MD, MHS[1]; Heather Hampel, MS[5]; Tasleem J. Padamsee, Ph D[6]; Michael J. Bishop, MD[7]; James J Kim, MD[8]; Shelly R. Hovick, Ph D[9]; William J. Irvin, Jr., MD[10]; Leigha Senter, MS[3+]; Daniel G. Stover, MD[1,2,11+]

POINT-BY-POINT RESPONSE TO REVIEWS

Review Comments to the Author

Reviewer #1: This is an original report describing the effect of a patient-facing educational video on patient understanding of tumor genomic testing. The rationale for the work is well described as is the sample frame.

There are several key points and questions for the authors:
1. How was the video content developed and appraised before deployment. That is, were patients involved in the creation of the video and pilot testing? What was the literacy level of the video?

As noted in the introduction, "*Based on data obtained through conducting diverse patient focus groups, we developed an animated 4-minute video for patient TGT education that addresses national guidelines surrounding TGT from the American Society of Clinical Oncology (ASCO) and the American College of Medical genetics and Genomics (ACMG)*."

Further detail regarding video development methods is described in the related publication that accompanied this submission: Senter, et al. "Patient Understanding of Tumor Genomic Testing: A Quality Improvement Effort." *JCO Oncology Practice* 2023. To avoid redundancy, we did not repeat the same text within this manuscript but cited it and included in the submission. A selection of the text from an additional manuscript (Veney DJ, et al *Cancer Med* 2024) is provided here:
"A provider QI survey highlighted inconsistency in pre-TGT discussion practice across providers; and patient focus groups and interviews revealed important themes and opportunities. Themes and opportunities were incorporated into a patient- navigated, concise 3–4 min animated video for pre- TGT education with content addressing 14/17 (82%) of key points described in the ASCO and ACMG guidelines. The video is based on adult learning and communication theory and includes characters of varied races, ethnicities, and genders so that the images presented are relatable to patients of varied identities."

If requested by the editorial team/reviewers we are happy to expand on the text within this manuscript but typically cite previously published studies rather than replicate text.

2. How were the novel survey instruments developed? Did the authors perform cognitive testing to determine whether the responses were measuring what they hoped to measure?

As noted in the Methods / Survey Instruments section, "*Video message–specific knowledge (VMSK): Our primary endpoint, video message-specific knowledge, was measured by 10 true/false statements that addressed key knowledge domains in the video intervention, with a final score reported as the number correct out of total (reported as percentage correct). Examples of statements included "The information that I get from tumor tissue genomic testing could be valuable to my children and other family members" and "I must have tumor genomic testing to continue with cancer treatment*.""

These questions were designed to specifically address the script of the video and ACMG/ASCO recommendations.

3. The authors describe the improvement in knowledge as "durable." How much time elapsed before the results of tumor genomic testing were provided to the patients? The average time in practice is 14 to 21 days. This reviewer is not convinced that this is a durable change in knowledge.

Our endpoint was dependent on the time lapse between testing and provider discussion of result with patient. Within the realities of cancer care such as missed appointments, hospitalizations, failed tests that required repeating, the actual time lapse between T1-T3 was an average of 50.58-days and a median of 42-days.

4. It does not appear that there was an a priori sample size determination. This is unusual for a project with a defined endpoint.

A power analysis was conducted for our primary endpoint and we have included the following text in the 'Statistical Analysis' section of Methods:
"*Power analysis was conducted for the primary endpoint of video message-specific knowledge/recall accuracy, measured as number correct out of 10 questions. Based on preliminary data, with a cohort of 50 patients, there would be 90% power to detect an effect size of 0.47 in change of recall accuracy from pre- (immediately prior) to post- (immediately after) video intervention, using a two-sided Wilcoxon signed-rank test with alpha of 0.05*."

5. One motivation for this work is to increase trust in clinicians. Do the authors have thoughts about the ability of a video to enhance trust?

Based on our prior work, we were indeed driven to investigate if this video intervention enhanced trust. *Our expectation was that with education regarding the limitations of TGT, trust might be persevered despite uninformative test results.* Contradictory to our hypothesis, there was no significant change (increase or decrease) in trust based on the 11-question Trust in Physician/Provider (TIPP) score. Specific statements from Results section:
"*There were no other significant associations between other patient characteristics and video message-specific knowledge, genomic knowledge/understanding or any patient characteristics and Trust in Physician/Provider*."
"*Despite significant change in VMSK over time, there was no significant change in GKU or TIPP for the overall population or any individual cohort (**Table 3**), reinforcing specific improvement seen only in VMSK appears likely to be a product of video viewing*."

6. The information on germline testing, while interesting, is distracting and detracts from the message of the paper. Since clinicians are the major barrier to genetic testing, it is not clear the mechanism by which improving tumor genomic testing would close the gap in genetic testing and counseling.

The potential for incidental germline findings is a critical component of national guidelines from both ACMG and ASCO. As such, a key goal of the video was to address guideline-concordant care, we included this although it was an exploratory analysis: *“As an exploratory endpoint, we investigated the incidental germline frequency and subsequent referrals for genetic counseling.” In the vein of exploratory analysis in general, this may be merely hypothesis generating for future research.*

6. What thoughts do the authors have about improving high quality information support to people with lower levels of education and/or health literacy? Without paying attention to those people with lower levels of education, we run the risk of creating even bigger gaps between the haves and have-nots.
We completely agree with this reviewer and it was precisely this concern that led us to add a cohort outside of our academic medical center:
“The addition of a community-centered cohort was intended to increase the heterogeneity of our participant population, not only geographically, but across all directly measured demographics such as race, ethnicity, education, and income, as well as through less easily defined social and cultural parameters.”
“Differences related to TGT application have likely narrowed in recent years with more broad uptake of TGT at all care sites,[13-17] while differences between community and academic patient populations, such as degree of education and income, have likely remained static due to barriers to care.”

7. Minor point: The patient flow diagram is not really a CONSORT diagram
This has been renamed ‘Patient Flow Diagram’ in Supp Fig 1 legend.

8. Minor point: There is a missing word in the Introduction in the sentence that begins: "While...application of TGT in clinical practice."
Corrected

Reviewer #2: Veney, et al explores an important issue – namely patient education for tumor genomic testing (TGT). Use of TGT is rapidly rising, however pre-testing education is often absent. Thus, key issues that drastically affect patient satisfaction, e.g. the possibility of detection of germline mutations and the fact that most often TGT does not lead to mutation-matched targeted therapy, can come as a surprise the patient after results are in-hand. The authors performed a prospective multicenter study assessing the impact of a 4 minute educational video on patient knowledge about TGT and its care implications. Overall, this is a well-conceived and implemented study addressing an important gap in precision oncology. I recommend acceptance with minor revision as detailed below.

Thank you – we appreciate the positive comments.

Minor points

Line 50. “data are available upon request..” Elsewhere is stated “all data are freely available..” These are different statements. I will leave to PLOS ONE editorial staff if these statements are adequately consistent with each other and with the journal’s data availability criteria for clinical trials. Ideally the authors will deposit the deidentified primary data in a repository.

The data for this study, now completed, are available on ClinicalTrials.gov we have updated the Data Availability statement to now read, **"***Raw data were generated at The Ohio State University. Data supporting the findings of this study are publicly available at ClinicalTrials.gov https://clinicaltrials.gov/study/NCT05215769.*"

The authors should clarify whether / to what degree the VMSK survey was validated and pilot tested.

As noted above, in the Methods / Survey Instruments section, "*Video message–specific knowledge (VMSK): Our primary endpoint, video message-specific knowledge, was measured by 10 true/false statements that addressed key knowledge domains in the video intervention, with a final score reported as the number correct out of total (reported as percentage correct). Examples of statements included "The information that I get from tumor tissue genomic testing could be valuable to my children and other family members" and "I must have tumor genomic testing to continue with cancer treatment*.""

These questions were designed to specifically address the script of the video and ACMG/ASCO recommendations.

How were ceiling effects handled given the high baseline scores on the VMSK?

Thanks to the reviewer for pointing these out. Because the data were highly skewed (even after log transformation), the Friedman test (a non-parametric test for repeated measures) was used to assess differences over time. The 'Statistical Analysis' section was updated to include the reasoning behind the Friedman test use: "*Survey measure change over time (T1:T2:T3) was evaluated by Friedman test, a non-parametric test for repeated measures because the data were highly skewed (even after log transformation)*."

Authors note only that 203 participants completed all 3 surveys, however authors should clarify attrition rates, how many participants were approached, how many participants were excluded from analysis, etc. This is important relative to CONSORT reporting.

Thank you – a 'patient flow diagram' (previously termed CONSORT diagram) is available in **Supplementary Figure 1A.** This addresses the 243 participants who signed informed consent and those that were excluded from analysis and reasons for exclusion.

Inasmuch as the cohort was mainly white, educated, and married, discussion should address implications for generalizability.

We agree and the following element was added to the Discussion, which now reads: "*An important limitation of this study is that participants were mainly white, educated, and married. The addition of a community-centered cohort was intended to increase the heterogeneity of our participant population, not only geographically, but across all directly measured demographics such as race, ethnicity, education, and income, as well as through less easily defined social and cultural parameters*."

Consistency between TGT being defined as "tissue genomic testing" or "tumor genomic testing" throughout is needed:
'Tissue genomic testing' reflects a statement made within the video so is only included in reference to that.

Line 161: (KSI)) should be (KSI) corrected
Corrected

Line 167: theright should be the right corrected
Corrected

Line 175: (OC).; should be (OC); corrected
Corrected

Reviewer #3: The authors present their analysis of survey responses from cancer patients undergoing tumor genomic testing who watched an informational video. The authors have previously published data on survey responses to this video from 150 patients recruited through Ohio State University (Cancer Med 2024)--for this analysis they added approximately 50 subjects recruited through community oncology clinics, and compared survey responses measured at 2 additional timepoints. They found the video increased knowledge scores for information presented in the video, but did not significantly increase scores for general genetics knowledge or trust in provider. Interesting findings included the fact that metastatic breast cancer patients appeared to have more familiarity with genomic tumor testing, particularly those with higher income and level of education. It is not clear what proportion of eligible individuals undergoing tumor testing viewed the video and/or completed the survey--or whether their demographics differed from study participants (88% of participants were white).

a 'patient flow diagram' (previously termed CONSORT diagram) is available in **Supplementary Figure 1A.** This addresses the 243 participants who signed informed consent and those that were excluded from analysis and reasons for exclusion

It would be interesting to include subgroup analyses of participants whose scores showed greatest improvement (vs least improvement) to identify additional specific interventions to improve information delivery to specific patient populations.

We agree.

Additional comments:
1. Introduction: Intro is very similar to authors' prior publication in Cancer Med 2024. The sentence structure (passive voice, run on sentences) and multiple abbreviations are a bit awkward and could benefit from editing.

We have revised the introduction to reduce passive voice, limit run-on sentences, and limit abbreviations as suggested.

2. Intro refers to "ethical challenges" in tumor genomic testing--perhaps worth elaborating further whether these relate to informed consent, understanding of results or differences in access to precision testing/ treatments.

We agree that the "ethical challenges" is a vague statement and did not significantly contribute to the manuscript so have removed this section to avoid confusion.

3.Methods: The number of clinics and demographics of the Bon Secours community sites are not described in detail

We have added the following statement to the 'Study Procedures', "*Participants at BSMH were seen in general oncology clinics at two sites in Ohio and one site in Virginia*."

Participant demographics for the BSMH cohort are presented in **Table 1. Patient Characteristics.**

4. Results: The differences between the breast cancer OSU patients and other cancer patients seem quite striking--were the breast cancer patients in the community settings as familiar with tumor testing or are the differences in knowledge primarily by socioeconomic status?

We agree with the reviewer that these differences were striking. The majority of the BSMH patients did not have breast cancer and the limited number of patients with breast cancer made broad conclusions difficult to discern but we hope to investigate this further as we roll this video out in the future.
