# Supplementary material for: Application of a concise video to improve patient understanding of tumor genomic testing in community and academic practice settings
Source: PLoS One. 2026 Oct 13;21(10):e0360611. doi: 10.1371/journal.pone.0360611 (PMC13660886; doi:10.1371/journal.pone.0360611)
Supplement: Supplementary file 7 [file pone.0360611.s008.docx]

**Application of a Concise Video to Improve Patient Understanding of Tumor Genomic Testing in Community and Academic Practice Settings**

Deloris J. Veney MACPR^1^, Lai Wei, PhD, MS^2^; Jaden Miller^3^; Amanda E. Toland, Ph D^3,4^; Carolyn J. Presley, MD, MHS^1^; Heather Hampel, MS^5^; Tasleem J. Padamsee, Ph D^6^; Michael J. Bishop, MD^7^; James J Kim, MD^8^; Shelly R. Hovick, Ph D^9^; William J. Irvin, Jr., MD^10^; Leigha Senter, MS^3+^; Daniel G. Stover, MD^1,2,11+^

**POINT-BY-POINT RESPONSE TO REVIEWS**

**Editorial Comments**

**1. Please ensure that your manuscript meets PLOS ONE's style requirements, including those for file naming.**

Confirmed

**2. We note that the grant information you provided in the ‘Funding Information’ and ‘Financial Disclosure’ sections do not match.
When you resubmit, please ensure that you provide the correct grant numbers for the awards you received for your study in the ‘Funding Information’ section.**

Thank you – we have reconciled this (below)

**3. Thank you for stating the following in the Acknowledgments Section of your manuscript:
“This work was supported by National Institutes of Health (NIH) grant 1R21CA259985 (LW, LS, SRH, HLH, AET, CJP, DGS), P30CA016058 (DGS, LW, TS, AET), 1R01 CA215151 (AET), CJP is supported by 1K76AG074923-01.”
We note that you have provided funding information that is not currently declared in your Funding Statement. However, funding information should not appear in the Acknowledgments section or other areas of your manuscript. We will only publish funding information present in the Funding Statement section of the online submission form.

Please remove any funding-related text from the manuscript and let us know how you would like to update your Funding Statement. Currently, your Funding Statement reads as follows:

“This work was supported by National Institutes of Health (NIH) grant 1R21CA259985 (LW, LS, SRH, HLH, AET, CJP, DGS); HealthyState Alliance Momentum Grant (DGS); National Institutes of Health (NIH) grant P30CA016058 (DGS, LW, TS, AET), National Institutes of Health (NIH) grant 1R01 CA215151 (AET) National Institutes of Health (NIH) grant to CJP is supported by 1K76AG074923-01. The sponsors or funders play any role in the study design, data collection and analysis, decision to publish, or preparation of the manuscript.** [**http://www.cancer.gov**](https://urldefense.com/v3/__http:/www.cancer.gov__;!!AU3bcTlGKuA!A21QZgE9zZWXOj8D6KJiM6UWeCPPe8WrjDt7vp_BsTYZQ9S2ZQ_1t0VeIenvtE8OuQaYd8N2nYnMHpRV_h_dL6zEQjLBuIk$)**”

Please include your amended statements within your cover letter; we will change the online submission form on your behalf.**

Thank you. We have removed it from the Acknowledgements and reconciled. The amended Funding Statement is:

“*This work was supported by National Institutes of Health (NIH) grant 1R21CA259985 (LW, LS, SRH, HLH, AET, CJP, DGS); HealthyState Alliance Momentum Grant (DGS); National Institutes of Health (NIH) grant P30CA016058 (DGS, LW, TS, AET); National Institutes of Health (NIH) grant 1R01CA215151 (AET); National Institutes of Health (NIH) grant 1K76AG074923 (CJP).*

*The sponsors or funders did not play any role in the study design, data collection and analysis, decision to publish, or preparation of the manuscript.*”

**4. Thank you for stating the following in the Competing Interests section:
“HH is on the scientific advisory board for Invitae Genetics, Genome Medical, and Promega. She has stock/stock options in Genome Medical and GI OnDemand. LS is a consultant and speaker for AstraZeneca. DS served on an advisory board for Novartis, Guardant, and receives research funding (in kind to institution) from Foundation Medicine and NeoGenomics. CJP received payment for patient education material development through Jazz Pharmaceuticals. ”

We note that you received funding from a commercial sources:
• AstraZeneca
• Novartis
• Guardant
• NeoGenomics
• Jazz Pharmaceuticals

Please provide an amended Competing Interests Statement that explicitly states this commercial funder, along with any other relevant declarations relating to employment, consultancy, patents, products in development, marketed products, etc.

Within this Competing Interests Statement, please confirm that this does not alter your adherence to all PLOS ONE policies on sharing data and materials by including the following statement: "This does not alter our adherence to PLOS ONE policies on sharing data and materials.” (as detailed online in our guide for authors** [**http://journals.plos.org/plosone/s/competing-interests**](https://urldefense.com/v3/__http:/journals.plos.org/plosone/s/competing-interests__;!!AU3bcTlGKuA!A21QZgE9zZWXOj8D6KJiM6UWeCPPe8WrjDt7vp_BsTYZQ9S2ZQ_1t0VeIenvtE8OuQaYd8N2nYnMHpRV_h_dL6zE_r1x5_Q$)**). If there are restrictions on sharing of data and/or materials, please state these. Please note that we cannot proceed with consideration of your article until this information has been declared.

Please include your amended Competing Interests Statement within your cover letter. We will change the online submission form on your behalf.**

The amended Competing Interests Statement now reads:

“*HH is on the scientific advisory board for Invitae Genetics, Genome Medical, and Promega. She has stock/stock options in Genome Medical and GI OnDemand. LS is a consultant and speaker for AstraZeneca. DS served on an advisory board for Novartis, Guardant, and receives research funding (in kind to institution) from Foundation Medicine and NeoGenomics. CJP received payment for patient education material development through Jazz Pharmaceuticals*. *This does not alter our adherence to PLOS ONE policies on sharing data and materials.”*

**5. Please provide a complete Data Availability Statement in the submission form, ensuring you include all necessary access information or a reason for why you are unable to make your data freely accessible. If your research concerns only data provided within your submission, please write "All data are in the manuscript and/or supporting information files" as your Data Availability Statement.**

Data Availability Statement updated in the submission form.

**6. Your ethics statement should only appear in the Methods section of your manuscript. If your ethics statement is written in any section besides the Methods, please move it to the Methods section and delete it from any other section. Please ensure that your ethics statement is included in your manuscript, as the ethics statement entered into the online submission form will not be published alongside your manuscript.**

This now only appears in the Methods.

**7. Please upload a new copy of Figures 1 a to 1d; and Supplementary Figure 1 as the detail is not clear. Please follow the link for more information:** [**https://journals.plos.org/plosone/s/figures**](https://urldefense.com/v3/__https:/journals.plos.org/plosone/s/figures__;!!AU3bcTlGKuA!A21QZgE9zZWXOj8D6KJiM6UWeCPPe8WrjDt7vp_BsTYZQ9S2ZQ_1t0VeIenvtE8OuQaYd8N2nYnMHpRV_h_dL6zEj_Fnixc$)

New copies of Figure 1 and Supplementary Figure 1 have been updated/uploaded

**8. Please include captions for your Supporting Information files at the end of your manuscript, and update any in-text citations to match accordingly. Please see our Supporting Information guidelines for more information:** [**http://journals.plos.org/plosone/s/supporting-information**](https://urldefense.com/v3/__http:/journals.plos.org/plosone/s/supporting-information__;!!AU3bcTlGKuA!A21QZgE9zZWXOj8D6KJiM6UWeCPPe8WrjDt7vp_BsTYZQ9S2ZQ_1t0VeIenvtE8OuQaYd8N2nYnMHpRV_h_dL6zEgfplm7Q$)**.**

Captions have been added for Supplementary figures and in-text citations updated.

**If the reviewer comments include a recommendation to cite specific previously published works, please review and evaluate these publications to determine whether they are relevant and should be cited. There is no requirement to cite these works unless the editor has indicated otherwise.** 

Noted.

**Additional Editor Comments (if provided):

The authors attempted to respond to previous comments yet the original concerns persist.
The authors were not sufficiently responsive to reviewers.

Please provide scientifically proofing and convincing point-by-point responses to the reviewers and revise the manuscript accordingly.**

Thank you – we believe that we have convincingly responded as below. Specifically, Reviewers 2-4 indicated “All comments have been addressed” and we responded in detail to Reviewer #1’s remaining critiques.

**Reviewers' comments:

Reviewer's Responses to Questions**

**Comments to the Author

1. If the authors have adequately addressed your comments raised in a previous round of review and you feel that this manuscript is now acceptable for publication, you may indicate that here to bypass the “Comments to the Author” section, enter your conflict of interest statement in the “Confidential to Editor” section, and submit your "Accept" recommendation.**

**Reviewer #1: (No Response)**

**Reviewer #2: All comments have been addressed**

**Reviewer #3: All comments have been addressed**

**Reviewer #4: All comments have been addressed**

**2. Is the manuscript technically sound, and do the data support the conclusions?

The manuscript must describe a technically sound piece of scientific research with data that supports the conclusions. Experiments must have been conducted rigorously, with appropriate controls, replication, and sample sizes. The conclusions must be drawn appropriately based on the data presented.**

**Reviewer #1: Partly**

**Reviewer #2: Yes**

**Reviewer #3: (No Response)**

**Reviewer #4: Yes**

**3. Has the statistical analysis been performed appropriately and rigorously?**

**Reviewer #1: Yes**

**Reviewer #2: Yes**

**Reviewer #3: (No Response)**

**Reviewer #4: Yes**

**4. Have the authors made all data underlying the findings in their manuscript fully available?

The** [**PLOS Data policy**](http://track.editorialmanager.com/CL0/http:%2F%2Fwww.plosone.org%2Fstatic%2Fpolicies.action%23sharing/1/010f01a06bcf2888-630073c8-6b72-4661-bfaa-c45ea0007f50-000000/fqTahOiTN9mlveuloLusieieR_AxMNMYQmF1W6YfMxI=258) **requires authors to make all data underlying the findings described in their manuscript fully available without restriction, with rare exception (please refer to the Data Availability Statement in the manuscript PDF file). The data should be provided as part of the manuscript or its supporting information, or deposited to a public repository. For example, in addition to summary statistics, the data points behind means, medians and variance measures should be available. If there are restrictions on publicly sharing data—e.g. participant privacy or use of data from a third party—those must be specified.**

**Reviewer #1: Yes**

**Reviewer #2: Yes**

**Reviewer #3: (No Response)**

**Reviewer #4: Yes**

**5. Is the manuscript presented in an intelligible fashion and written in standard English?

PLOS ONE does not copyedit accepted manuscripts, so the language in submitted articles must be clear, correct, and unambiguous. Any typographical or grammatical errors should be corrected at revision, so please note any specific errors here.**

**Reviewer #1: Yes**

**Reviewer #2: Yes**

**Reviewer #3: (No Response)**

**Reviewer #4: Yes**

**6. Review Comments to the Author

Please use the space provided to explain your answers to the questions above. You may also include additional comments for the author, including concerns about dual publication, research ethics, or publication ethics. (Please upload your review as an attachment if it exceeds 20,000 characters)**

**Reviewer #1: The authors attempted to respond to previous comments yet the original concerns persist as follows:**

**1.. While developed with patient input, It does not appear that the video was viewed and evaluated by patients/caregivers before deployment having the same or additional patients evaluate the video would increase confidence that the video met the information needs it intended to meet.**

Thank you to the Reviewer for commenting on this point.

This video has NOT yet been deployed outside of a research setting.

The goal of this study is to have additional patients evaluate the video (in a research design) to confirm that the video meets the information needs it intends to meet. Thus, this is the study population of 203 participants viewing the video where we establish the video as an evidence-based intervention prior to clinical use.

In addition, the video was viewed by cancer care providers for various cancer types, presented at Grand Rounds at OSU and BSMH, and to nursing staff at all institutions. It was created as a collaboration between Genetic Counseling, Medical Oncology, and with the input of an adult health and risk communication expert.

We did not hold a second round of focus groups. Our original focus groups and interviewees were metastatic cancer patients. Due to the amount of time that it took to create the video and the reality of stage IV cancer it was not feasible to return to the same patients for additional input.

In the preliminary results, respondents confirmed that the video met the information needs:

“*A Likert scale of agreement was used to measure participant's opinions of the video's utility, clarity, and engagement (Figure* [***S4C***](https://onlinelibrary.wiley.com/doi/10.1002/cam4.70095?msockid=2414a38969cb6c21076eb44768b26d03#support-information-section)*), and most (>80%) of respondents agreed or strongly agreed that the video was helpful, easy to understand, and held their attention, with fewer but still most (60%) indicating that the graphics were helpful. Most participants (141 of 150) felt that the amount of information presented was adequate, while four participants felt the content was excessive, and five participants found the content insufficient*.”

-From: Veney DJ, Wei LY, Toland AE, et al. *A video intervention to improve patient understanding of tumor genomic testing in patients with cancer*. *Cancer Med*. 2024; 13:e70095. doi:[10.1002/cam4.70095](https://doi.org/10.1002/cam4.70095)

**2. The VMSK does not appear to have been validated. The authors do a good job explaining where the items originated, there is no description of cognitive testing (in which people not involved in survey development respond in detail to what they think they are being asked). This is how one confirms that the instrument is measuring what it is designed to measure. It does not appear that the instrument has been validated. Validation requires several steps, including comparing responses to other published instruments to see if responses are concordant with (at least in some aspect) with previously-used and validated instruments.**

We agree with the reviewer regarding the importance of using validated instruments and employed validated instruments whenever possible, including GKU, TIPP, and knowledge and knowledge insufficiency from the Risk Information Seeking and Processing Model.

There was no previously validated measure to directly assess knowledge change obtained through our video intervention. The initial development of the True/False VMSK items, taken directly from the video script, were grounded in preliminary quality improvement work, including patient focus groups, qualitative interviews, and provider surveys, to ensure content validity and clear understanding of the core concepts prior to research instrument deployment at OSU. This is published work: Senter L, Veney D, Surplus T, et al. Patient Understanding of Tumor Genomic Testing: A Quality Improvement Effort. *JCO Oncol Pract*. 2023;19(1):e8-e14. doi:10.1200/OP.22.00316.

We agree with the reviewer that comparison to validated instruments is critical and, as part of the study design, the VMSK and the validated GKU instruments were administered simultaneously to all participants at T1 and T2. This dual-question design was intended to allow us to demonstrate concordance at T1, and by repeating both GKU and VMSK at T2, also allow us to attribute pre-test/post-test score change in VMSK solely to the video Intervention.

While participants demonstrated a statistically significant increase in VMSK scores following the educational intervention (median increase of 10 points; $p<0.0001$), their scores on the published GKU survey remained completely unchanged pre- to post-video ($p=0.89$). This demonstrated both convergent validity, the VMSK sensitive to message-specific knowledge gain, and construct validity, the intervention specifically improved knowledge related to the video content rather than triggering generalized genomic literacy changes.

“**As an internal control, genomic knowledge/understanding did not significantly change suggesting that knowledge gained was specifically related to the video.”**

We have now revised the Discussion section of the manuscript submission to read.

“VMSK provides insight into the key material specifically included in the video, while GKU serves as a previously validated baseline measure and concurrent benchmark (alongside the VMSK items) to evaluate participant performance. The absence of a previously-validated survey instrument that directly addresses tumor genomic testing is a limitation to this study because individual question response concordance could not be directly assessed. Knowledge concordance between VMSK and GKU provided a surrogate measure for VMSK instrument validation purposes.”

In summary, by using this combination of previously validated instruments in conjunction with our VMSK instrument, we simultaneously use the validated instruments to gather data on the video intervention and provide validity for the VMSK survey.

**3. The authors concur that attending to the needs of people with lower levels of education is an important area to address. There is nothing in the revised manuscript that attends to this concern other than reiterating the intention of including community practices. This reviewer would encourage the authors to not only expand their sample but also to consult with and incorporate the views and comprehension of this important target population through iterative work with their patient community.**

Thank you to the reviewer for this explanation of their concern. As noted, in **Table 1**, we demonstrate that through the addition of the Community cohort, we added to the demographic divisions that had lower levels of education: those categories that represent individuals that did not graduate high school and those whose formal education terminated at high school graduation. High school graduates are the majority of this cohort (versus our other three cohorts where the majority of respondents claimed education beyond high school).

We also comment within the manuscript “Interestingly, while the T1:T2 absolute improvement was the greatest in Cohort 1: Breast, the T1:T3 absolute improvement was greatest in Cohort 4: Community (**Fig 1A**), suggesting that different groups *(for example, those with distinct educational attainment)* may benefit and retain the information differently.”

The addition of the Community cohort, a central element of this study, represents the iterative work that the reviewer suggests. We certainly saw that across all 203 participants, those with lowest education attainment demonstrated lowest T1 VMSK scores and shallowest knowledge gains, approximately 7 pts vs 9 pts for all other brackets. We presented this in the **Results** section:

*Association of Baseline Patient Characteristics with Survey Outcomes*

The association of patient characteristics with baseline VMSK, GKU, and TIPP were assessed via Spearman correlations (**Table 2**). Across the entire study population there was a significant association between education and baseline/T1 VMSK (nominal p<0.0001) and GKU (nom p=0.004) as well as income and baseline/T1 GKU (nom p=0.007) and GKU (nom p=0.004).

Because we saw knowledge gains across all educational divisions, we were confident that despite the T1/T2 12-pt+ score separations between participants with least-to-most formal education, all cohorts would receive benefit from video viewing. The score changes were consistent with our previous analysis so we focused on the new information from the community cohort: their knowledge recall was the most improved. The following statement was added to the **Results** section “Mean VMSK score gains across the educational demographic divisions of some high school education or less, high school graduate or equivalent, some higher education, and college graduate were 7.14, 9.05, 7.09, and 11.08 respectively.” And “With all education demographic divisions showing VMSK score improvement, we demonstrate that the video intervention provides an educational resource for learners of varied educational attainment and does not negatively impact comprehension surrounding TGT for any bracket.” was added to the **Discussion** section.

Mean VMSK Scores by Education n=203

|  | SomeHSorLess | HSGraduate | SomeHigherEd | CollegeGrad |
| --- | --- | --- | --- | --- |
| T1mean | 75.7143 | 84.0476 | 88.2558 | 86.25 |
| T2mean | 82.8571 | 93.0952 | 95.3488 | 97.3333 |

**(Reviewer #2: Authors have adequately responded to queries of all three reviewers and so i see no barriers to acceptance.**

**Reviewer #3: (No Response)**

**Reviewer #4:**

**Dear Authors,**

**This a well-executed manuscript evaluating the impact of a brief educational intervention designed to improve patient understanding of tumor genomic testing (TGT). The main findings demonstrate that this video significantly improved patient knowledge related to the specific educational messages conveyed in the video, which were maintained through the return of TGT results. The study addresses an important gap by assessing both the immediate and longer-term effects of a concise educational video across multiple cancer types and practice settings.**

**Notably, while video message-specific knowledge improved significantly, objective genomic knowledge and trust in providers remained unchanged, suggesting that the intervention was effective in communicating key practical concepts but did not have an effect on broader genomic literacy or patient-provider trust.**

**Overall, this study provides important evidence supporting the use of brief, standardized educational materials to enhance patient understanding of TGT and may serve as a model for improving implementation of genomic medicine in diverse clinical settings.**

**Overall, this manuscript in its current iteration has addressed all of the important issues raised by the previous reviews. Therefore, I have minor edits/suggestions at this time:**

**1. Line 88: The field is moving away from using the terminology "mutation", the authors should consider alternatives such as "targeted therapies guided by genomic alterations", "genomically guided therapies" or "molecularly targeted therapies"**

Thank you to the reviewer for this insight and for the rephrasing suggestions. These changes have been incorporated from lines 84-90.

**2. Lines 115-116: Please rephrase for clarity, "…,some specifically do not want testing". Do the authors mean that patients wants to be notified but do not want to pursue confirmatory germline testing? Or some want to be notified of secondary findings but some do not?**

Thank you to the Reviewer for pointing out where clarity was needed. In fact, the three publications cited in regard to this statement reflect that some patients simply do not want any genomic testing while others do not want follow-up testing for incidental findings. The reasons for this remain unclear and it was our hope that with education, patients would see the value of testing and avoid confusion with genetic testing. This sentence now reads “*some patients do not want initial genomic testing and/or follow-up genetic testing if incidental findings are discovered*.”

**3. In relation to reviewer 1, comment 3 can the authors add some detail on how they determined that the TGT was discussed during the visit in order to administer T3? As we know, TGT results are not always discussed and if the TGT was uninformative, they may have spend follow-up appointments on other items.**

Thank you to the reviewer for this question. An early obstacle to study performance was the lack of provider follow-up of TGT results. It is one element that affects the duration of time between T1/T2 -T3. The following addition has been made to the Methods section of the manuscript “*Study personnel were embedded in clinic teams to remind providers to discuss the results of TGT with patients and to verify that those discussions took place prior to participants being requested to complete the T3 survey. Participants were only offered the T3 survey if chart documentation was present that indicated that TGT results were communicated to the patient or if the Provider verbally confirmed with the survey administrator that the requisite conversations took place*.”

**4. Do the authors have any insight into why those who had highly penetrant suspected germline findings were not referred?**

Thank you to the Reviewer for posing this important question. This was investigated separately at OSU through quality improvement data gathering where the rate of omission was similar (abstract in preparation), suggesting that this phenomenon was not limited to the current study population. Efforts to improve that specific metric are being handled within that same QI endeavor. We hypothesize that this may be a combination of factors, including provider knowledge, patient awareness, and time in clinic – among other potential factors. In the absence of firm data, we noted, “*Our prior work suggests that incidental germline findings are not discussed routinely in oncology care largely due to time constraints*.“

**5. Line 300-301: The option to opt-out of germline results is not always available and will depend on TGT vendor and state regulations. Please change to "reviewing potential options to opt-out"**

Thank you to the Reviewer for volunteering this information and corrective phrasing. This suggestion has been incorporated in the manuscript. Line 300-301, now line 309-311, reads “*These organizations echo the importance of informed consent prior to TGT and reviewing potential options for patients to opt out of receipt of germline results*.”

**Thank you for inviting me to review this interesting study.**
