# Supplementary material for: Application of a concise video to improve patient understanding of tumor genomic testing in community and academic practice settings
Source: PLoS One. 2026 Oct 13;21(10):e0360611. doi: 10.1371/journal.pone.0360611 (PMC13660886; doi:10.1371/journal.pone.0360611)
Supplement: Peer review history

# Author comment: Author response to Decision Letter 0

**DOI:** 10.1371/journal.pone.0360611.r001

**Transfer Alert**

This paper was transferred from another journal. As a result, its full editorial history (including decision letters, peer reviews and author responses) may not be present.

19 Mar 2026

# Peer review: Decision Letter 0

**DOI:** 10.1371/journal.pone.0360611.r002

**Authors:** James Maloney

28 May 2026

PONE-D-26-13280

Application of a Concise Video to Improve Patient Understanding of Tumor Genomic Testing in Community and Academic Practice Settings

PLOS One

Dear Dr. Stover,

Thank you for submitting your manuscript to PLOS ONE. After careful consideration, we have decided that your manuscript does not meet our criteria for publication and must therefore be rejected.

Specifically:

The reviewers felt that this project had a number of limitations that are well outlined by their comments. These limitations are substantial enough to preclude a major revision approach, so the manuscript is rejected. We hope these comments will help the authors revise their work for a future submission to another journal.

I am sorry that we cannot be more positive on this occasion, but hope that you appreciate the reasons for this decision.

Kind regards,

James P. Maloney, MD

Academic Editor

PLOS One

Reviewers' comments:

Reviewer's Responses to Questions

**Comments to the Author**

1. Is the manuscript technically sound, and do the data support the conclusions?

Reviewer #1: Partly

Reviewer #2: Yes

Reviewer #3: Yes

**********

2. Has the statistical analysis been performed appropriately and rigorously?

Reviewer #1: No

Reviewer #2: Yes

Reviewer #3: Yes

**********

3. Have the authors made all data underlying the findings in their manuscript fully available?

The PLOS Data policy

Reviewer #1: Yes

Reviewer #2: No

Reviewer #3: Yes

**********

4. Is the manuscript presented in an intelligible fashion and written in standard English?

Reviewer #1: Yes

Reviewer #2: Yes

Reviewer #3: Yes

**********

Reviewer #1: This is an original report describing the effect of a patient-facing educational video on patient understanding of tumor genomic testing. The rationale for the work is well described as is the sample frame.

There are several key points and questions for the authors:

1. How was the video content developed and appraised before deployment. That is, were patients involved in the creation of the video and pilot testing? What was the literacy level of the video?

2. How were the novel survey instruments developed? Did the authors perform cognitive testing to determine whether the responses were measuring what they hoped to measure?

3. The authors describe the improvement in knowledge as "durable." How much time elapsed before the results of tumor genomic testing were provided to the patients? The average time in practice is 14 to 21 days. This reviewer is not convinced that this is a durable change in knowledge.

4. It does not appear that there was an a priori sample size determination. This is unusual for a project with a defined endpoint.

5. One motivation for this work is to increase trust in clinicians. Do the authors have thoughts about the ability of a video to enhance trust?

6. The information on germline testing, while interesting, is distracting and detracts from the message of the paper. Since clinicians are the major barrier to genetic testing, it is not clear the mechanism by which improving tumor genomic testing would close the gap in genetic testing and counseling.

6. What thoughts do the authors have about improving high quality information support to people with lower levels of education and/or health literacy? Without paying attention to those people with lower levels of education, we run the risk of creating even bigger gaps between the haves and have-nots.

7. Minor point: The patient flow diagram is not really a CONSORT diagram

8. Minor point: There is a missing word in the Introduction in the sentence that begins: "While...application of TGT in clinical practice."

Reviewer #2: Veney, et al explores an important issue – namely patient education for tumor genomic testing (TGT). Use of TGT is rapidly rising, however pre-testing education is often absent. Thus, key issues that drastically affect patient satisfaction, e.g. the possibility of detection of germline mutations and the fact that most often TGT does not lead to mutation-matched targeted therapy, can come as a surprise the patient after results are in-hand. The authors performed a prospective multicenter study assessing the impact of a 4 minute educational video on patient knowledge about TGT and its care implications. Overall, this is a well-conceived and implemented study addressing an important gap in precision oncology. I recommend acceptance with minor revision as detailed below.

Minor points

Line 50. “data are available upon request..” Elsewhere is stated “all data are freely available..” These are different statements. I will leave to PLOS ONE editorial staff if these statements are adequately consistent with each other and with the journal’s data availability criteria for clinical trials. Ideally the authors will deposit the deidentified primary data in a repository.

The authors should clarify whether / to what degree the VMSK survey was validated and pilot tested.

How were ceiling effects handled given the high baseline scores on the VMSK?

Authors note only that 203 participants completed all 3 surveys, however authors should clarify attrition rates, how many participants were approached, how many participants were excluded from analysis, etc. This is important relative to CONSORT reporting.

Inasmuch as the cohort was mainly white, educated, and married, discussion should address implications for generalizability.

Consistency between TGT being defined as “tissue genomic testing” or “tumor genomic testing” throughout is needed

Line 161: (KSI)) should be (KSI)

Line 167: theright should be the right

Line 175: (OC).; should be (OC);

Reviewer #3: The authors present their analysis of survey responses from cancer patients undergoing tumor genomic testing who watched an informational video. The authors have previously published data on survey responses to this video from 150 patients recruited through Ohio State University (Cancer Med 2024)--for this analysis they added approximately 50 subjects recruited through community oncology clinics, and compared survey responses measured at 2 additional timepoints. They found the video increased knowledge scores for information presented in the video, but did not significantly increase scores for general genetics knowledge or trust in provider. Interesting findings included the fact that metastatic breast cancer patients appeared to have more familiarity with genomic tumor testing, particularly those with higher income and level of education. It is not clear what proportion of eligible individuals undergoing tumor testing viewed the video and/or completed the survey--or whether their demographics differed from study participants (88% of participants were white). It would be interesting to include subgroup analyses of participants whose scores showed greatest improvement (vs least improvement) to identify additional specific interventions to improve information delivery to specific patient populations.

Additional comments:

1. Introduction: Intro is very similar to authors' prior publication in Cancer Med 2024. The sentence structure (passive voice, run on sentences) and multiple abbreviations are a bit awkward and could benefit from editing.

2. Intro refers to "ethical challenges" in tumor genomic testing--perhaps worth elaborating further whether these relate to informed consent, understanding of results or differences in access to precision testing/ treatments.

3.Methods: The number of clinics and demographics of the Bon Secours community sites are not described in detail

4. Results: The differences between the breast cancer OSU patients and other cancer patients seem quite striking--were the breast cancer patients in the community settings as familiar with tumor testing or are the differences in knowledge primarily by socioeconomic status?

**********

what does this mean?). If published, this will include your full peer review and any attached files.

If you choose “no”, your identity will remain anonymous but your review may still be made public.

**Do you want your identity to be public for this peer review?**  For information about this choice, including consent withdrawal, please see our Privacy Policy

Reviewer #1: No

Reviewer #2: No

Reviewer #3: No

[NOTE: If reviewer comments were submitted as an attachment file, they will be attached to this email and accessible via the submission site. Please log into your account, locate the manuscript record, and check for the action link "View Attachments". If this link does not appear, there are no attachment files.]

- - - - -

For journal use only: PONEDEC3

# Author comment: Author response to Decision Letter 1

**DOI:** 10.1371/journal.pone.0360611.r003

16 Jul 2026

Application of a Concise Video to Improve Patient Understanding of Tumor Genomic Testing in Community and Academic Practice Settings

Deloris J. Veney MACPR1, Lai Wei, PhD, MS2; Jaden Miller3; Amanda E. Toland, Ph D3,4; Carolyn J. Presley, MD, MHS1; Heather Hampel, MS5; Tasleem J. Padamsee, Ph D6; Michael J. Bishop, MD7; James J Kim, MD8; Shelly R. Hovick, Ph D9; William J. Irvin, Jr., MD10; Leigha Senter, MS3+; Daniel G. Stover, MD1,2,11+

POINT-BY-POINT RESPONSE TO REVIEWS

Review Comments to the Author

Reviewer #1: This is an original report describing the effect of a patient-facing educational video on patient understanding of tumor genomic testing. The rationale for the work is well described as is the sample frame.

There are several key points and questions for the authors:

1. How was the video content developed and appraised before deployment. That is, were patients involved in the creation of the video and pilot testing? What was the literacy level of the video?

As noted in the introduction, “Based on data obtained through conducting diverse patient focus groups, we developed an animated 4-minute video for patient TGT education that addresses national guidelines surrounding TGT from the American Society of Clinical Oncology (ASCO) and the American College of Medical genetics and Genomics (ACMG).”

Further detail regarding video development methods is described in the related publication that accompanied this submission: Senter, et al. “Patient Understanding of Tumor Genomic Testing: A Quality Improvement Effort.” JCO Oncology Practice 2023. To avoid redundancy, we did not repeat the same text within this manuscript but cited it and included in the submission. A selection of the text from an additional manuscript (Veney DJ, et al Cancer Med 2024) is provided here:

“A provider QI survey highlighted inconsistency in pre-TGT discussion practice across providers; and patient focus groups and interviews revealed important themes and opportunities. Themes and opportunities were incorporated into a patient- navigated, concise 3–4 min animated video for pre- TGT education with content addressing 14/17 (82%) of key points described in the ASCO and ACMG guidelines. The video is based on adult learning and communication theory and includes characters of varied races, ethnicities, and genders so that the images presented are relatable to patients of varied identities.”

If requested by the editorial team/reviewers we are happy to expand on the text within this manuscript but typically cite previously published studies rather than replicate text.

2. How were the novel survey instruments developed? Did the authors perform cognitive testing to determine whether the responses were measuring what they hoped to measure?

As noted in the Methods / Survey Instruments section, “Video message–specific knowledge (VMSK): Our primary endpoint, video message-specific knowledge, was measured by 10 true/false statements that addressed key knowledge domains in the video intervention, with a final score reported as the number correct out of total (reported as percentage correct). Examples of statements included “The information that I get from tumor tissue genomic testing could be valuable to my children and other family members” and “I must have tumor genomic testing to continue with cancer treatment.””

These questions were designed to specifically address the script of the video and ACMG/ASCO recommendations.

3. The authors describe the improvement in knowledge as "durable." How much time elapsed before the results of tumor genomic testing were provided to the patients? The average time in practice is 14 to 21 days. This reviewer is not convinced that this is a durable change in knowledge.

Our endpoint was dependent on the time lapse between testing and provider discussion of result with patient. Within the realities of cancer care such as missed appointments, hospitalizations, failed tests that required repeating, the actual time lapse between T1-T3 was an average of 50.58-days and a median of 42-days.

4. It does not appear that there was an a priori sample size determination. This is unusual for a project with a defined endpoint.

A power analysis was conducted for our primary endpoint and we have included the following text in the ‘Statistical Analysis’ section of Methods:

“Power analysis was conducted for the primary endpoint of video message-specific knowledge/recall accuracy, measured as number correct out of 10 questions. Based on preliminary data, with a cohort of 50 patients, there would be 90% power to detect an effect size of 0.47 in change of recall accuracy from pre- (immediately prior) to post- (immediately after) video intervention, using a two-sided Wilcoxon signed-rank test with alpha of 0.05.”

5. One motivation for this work is to increase trust in clinicians. Do the authors have thoughts about the ability of a video to enhance trust?

Based on our prior work, we were indeed driven to investigate if this video intervention enhanced trust. Our expectation was that with education regarding the limitations of TGT, trust might be persevered despite uninformative test results. Contradictory to our hypothesis, there was no significant change (increase or decrease) in trust based on the 11-question Trust in Physician/Provider (TIPP) score. Specific statements from Results section:

“There were no other significant associations between other patient characteristics and video message-specific knowledge, genomic knowledge/understanding or any patient characteristics and Trust in Physician/Provider.”

“Despite significant change in VMSK over time, there was no significant change in GKU or TIPP for the overall population or any individual cohort (Table 3), reinforcing specific improvement seen only in VMSK appears likely to be a product of video viewing.”

6. The information on germline testing, while interesting, is distracting and detracts from the message of the paper. Since clinicians are the major barrier to genetic testing, it is not clear the mechanism by which improving tumor genomic testing would close the gap in genetic testing and counseling.

The potential for incidental germline findings is a critical component of national guidelines from both ACMG and ASCO. As such, a key goal of the video was to address guideline-concordant care, we included this although it was an exploratory analysis: “As an exploratory endpoint, we investigated the incidental germline frequency and subsequent referrals for genetic counseling.” In the vein of exploratory analysis in general, this may be merely hypothesis generating for future research.

6. What thoughts do the authors have about improving high quality information support to people with lower levels of education and/or health literacy? Without paying attention to those people with lower levels of education, we run the risk of creating even bigger gaps between the haves and have-nots.

We completely agree with this reviewer and it was precisely this concern that led us to add a cohort outside of our academic medical center:

“The addition of a community-centered cohort was intended to increase the heterogeneity of our participant population, not only geographically, but across all directly measured demographics such as race, ethnicity, education, and income, as well as through less easily defined social and cultural parameters.”

“Differences related to TGT application have likely narrowed in recent years with more broad uptake of TGT at all care sites,13-17 while differences between community and academic patient populations, such as degree of education and income, have likely remained static due to barriers to care.”

7. Minor point: The patient flow diagram is not really a CONSORT diagram

This has been renamed ‘Patient Flow Diagram’ in Supp Fig 1 legend.

8. Minor point: There is a missing word in the Introduction in the sentence that begins: "While...application of TGT in clinical practice."

Corrected

Reviewer #2: Veney, et al explores an important issue – namely patient education for tumor genomic testing (TGT). Use of TGT is rapidly rising, however pre-testing education is often absent. Thus, key issues that drastically affect patient satisfaction, e.g. the possibility of detection of germline mutations and the fact that most often TGT does not lead to mutation-matched targeted therapy, can come as a surprise the patient after results are in-hand. The authors performed a prospective multicenter study assessing the impact of a 4 minute educational video on patient knowledge about TGT and its care implications. Overall, this is a well-conceived and implemented study addressing an important gap in precision oncology. I recommend acceptance with minor revision as detailed below.

Thank you – we appreciate the positive comments.

Minor points

Line 50. “data are available upon request..” Elsewhere is stated “all data are freely available..” These are different statements. I will leave to PLOS ONE editorial staff if these statements are adequately consistent with each other and with the journal’s data availability criteria for clinical trials. Ideally the authors will deposit the deidentified primary data in a repository.

The data for this study, now completed, are available on ClinicalTrials.gov we have updated the Data Availability statement to now read, “Raw data were generated at The Ohio State University. Data supporting the findings of this study are publicly available at ClinicalTrials.gov https://clinicaltrials.gov/study/NCT05215769.”

The authors should clarify whether / to what degree the VMSK survey was validated and pilot tested.

As noted above, in the Methods / Survey Instruments section, “Video message–specific knowledge (VMSK): Our primary endpoint, video message-specific knowledge, was measured by 10 true/false statements that addressed key knowledge domains in the video intervention, with a final score reported as the number correct out of total (reported as percentage correct). Examples of statements included “The information that I get from tumor tissue genomic testing could be valuable to my children and other family members” and “I must have tumor genomic testing to continue with cancer treatment.””

These questions were designed to specifically address the script of the video and ACMG/ASCO recommendations.

How were ceiling effects handled given the high baseline scores on the VMSK?

Thanks to the reviewer for pointing these out. Because the data were highly skewed (even after log transformation), the Friedman test (a non-parametric test for repeated measures) was used to assess differences over time. The ‘Statistical Analysis’ section was updated to include the reasoning behind the Friedman test use: “Survey measure change over time (T1:T2:T3) was evaluated by Friedman test, a non-parametric test for repeated measures because the data were highly skewed (even after log transformation).”

Authors note only that 203 participants completed all 3 surveys, however authors should clarify attrition rates, how many participants were approached, how many participants were excluded from analysis, etc. This is important relative to CONSORT reporting.

Thank you – a ‘patient flow diagram’ (previously termed CONSORT diagram) is available in Supplementary Figure 1A. This addresses the 243 participants who signed informed consent and those that were excluded from analysis and reasons for exclusion.

Inasmuch as the cohort was mainly white, educated, and married, discussion should address implications for generalizability.

We agree and the following element was added to the Discussion, which now reads: “An important limitation of this study is that participants were mainly white, educated, and married. The addition of a community-centered cohort was intended to increase the heterogeneity of our participant population, not only geographically, but across all directly measured demographics such as race, ethnicity, education, and income, as well as through less easily defined social and cultural parameters.”

Consistency between TGT being defined as “tissue genomic testing” or “tumor genomic testing” throughout is needed:

‘Tissue genomic testing’ reflects a statement made within the video so is only included in reference to that.

Line 161: (KSI)) should be (KSI) corrected

Corrected

Line 167: theright should be the right corrected

Corrected

Line 175: (OC).; should be (OC); corrected

Corrected

Reviewer #3: The authors present their analysis of survey responses from cancer patients undergoing tumor genomic testing who watched an informational video. The authors have previously published data on survey responses to this video from 150 patients recruited through Ohio State University (Cancer Med 2024)--for this analysis they added approximately 50 subjects recruited through community oncology clinics, and compared survey responses measured at 2 additional timepoints. They found the video increased knowledge scores for information presented in the video, but did not significantly increase scores for general genetics knowledge or trust in provider. Interesting findings included the fact that metastatic breast cancer patients appeared to have more familiarity with genomic tumor testing, particularly those with higher income and level of education. It is not clear what proportion of eligible individuals undergoing tumor testing viewed the video and/or completed the survey--or whether their demographics differed from study participants (88% of participants were white).

a ‘patient flow diagram’ (previously termed CONSORT diagram) is available in Supplementary Figure 1A. This addresses the 243 participants who signed informed consent and those that were excluded from analysis and reasons for exclusion

It would be interesting to include subgroup analyses of participants whose scores showed greatest improvement (vs least improvement) to identify additional specific interventions to improve information delivery to specific patient populations.

We agree.

Additional comments:

1. Introduction: Intro is very similar to authors' prior publication in Cancer Med 2024. The sentence structure (passive voice, run on sentences) and multiple abbreviations are a bit awkward and could benefit from editing.

We have revised the introduction to reduce passive voice, limit run-on sentences, and limit abbreviations as suggested.

2. Intro refers to "ethical challenges" in tumor genomic testing--perhaps worth elaborating further whether these relate to informed consent, understanding of results or differences in access to precision testing/ treatments.

We agree that the “ethical challenges” is a vague statement and did not significantly contribute to the manuscript so have removed this section to avoid confusion.

3.Methods: The number of clinics and demographics of the Bon Secours community sites are not described in detail

We have added the following statement to the ‘Study Procedures’, “Participants at BSMH were seen in general oncology clinics at two sites in Ohio and one site in Virginia.”

Participant demographics for the BSMH cohort are presented in Table 1. Patient Characteristics.

4. Results: The differences between the breast cancer OSU patients and other cancer patients seem quite striking--were the breast cancer patients in the community settings as familiar with tumor testing or are the differences in knowledge primarily by socioeconomic status?

We agree with the reviewer that these differences were striking. The majority of the BSMH patients did not have breast cancer and the limited number of patients with breast cancer made broad conclusions difficult to discern but we hope to investigate this further as we roll this video out in the future.

Attachment Submitted filename: TGTVideo_ResponseToReviewers.pdf

# Peer review: Decision Letter 1

**DOI:** 10.1371/journal.pone.0360611.r004

**Authors:** Kokouvi Kassegne

4 Sep 2026

Dear Dr. Daniel Stover,

Thank you for submitting your manuscript to PLOS One. After careful consideration, we feel that it has merit but does not fully meet PLOS One’s publication criteria as it currently stands. Therefore, we invite you to submit a revised version of the manuscript that addresses the points raised during the review process.

Please submit your revised manuscript by 30 September 2026. If you will need more time than this to complete your revisions, please reply to this message or contact the journal office at plosone@plos.org. When you're ready to submit your revision, log on to https://www.editorialmanager.com/pone/ and select the 'Submissions Needing Revision' folder to locate your manuscript file.

- A letter that responds to each point raised by the academic editor and reviewer(s). You should upload this letter as a separate file labeled 'Response to Reviewers'.
- A marked-up copy of your manuscript that highlights changes made to the original version. You should upload this as a separate file labeled 'Revised Manuscript with Track Changes'.
- An unmarked version of your revised paper without tracked changes. You should upload this as a separate file labeled 'Manuscript'.

If you would like to make changes to your financial disclosure, please include your updated statement in your cover letter. Guidelines for resubmitting your figure files are available below the reviewer comments at the end of this letter.

If applicable, we recommend that you deposit your laboratory protocols in protocols.io to enhance the reproducibility of your results. Protocols.io assigns your protocol its own identifier (DOI) so that it can be cited independently in the future. For instructions see: https://journals.plos.org/plosone/s/submission-guidelines#loc-laboratory-protocols. Additionally, PLOS One offers an option for publishing peer-reviewed Lab Protocol articles, which describe protocols hosted on protocols.io. Read more information on sharing protocols at https://plos.org/protocols?utm_medium=editorial-email&utm_source=authorletters&utm_campaign=protocols.

As the corresponding author, your ORCID iD is verified in the submission system and will appear in the published article. PLOS supports the use of ORCID, and we encourage all coauthors to register for an ORCID iD and use it as well. Please encourage your coauthors to verify their ORCID iD within the submission system before final acceptance, as unverified ORCID iDs will not appear in the published article. *Only*  the individual author can complete the verification step; PLOS staff *cannot*  verify ORCID iDs on behalf of authors.

We look forward to receiving your revised manuscript.

Kind regards,

Kokouvi Kassegne

Academic Editor

PLOS One

Journal Requirements:

When submitting your revision, we need you to address these additional requirements.

1. Please ensure that your manuscript meets PLOS ONE's style requirements, including those for file naming. The PLOS ONE style templates can be found at

https://journals.plos.org/plosone/s/file?id=wjVg/PLOSOne_formatting_sample_main_body.pdf and

https://journals.plos.org/plosone/s/file?id=ba62/PLOSOne_formatting_sample_title_authors_affiliations.pdf

2. We note that the grant information you provided in the ‘Funding Information’ and ‘Financial Disclosure’ sections do not match.

When you resubmit, please ensure that you provide the correct grant numbers for the awards you received for your study in the ‘Funding Information’ section.

3. Thank you for stating the following in the Acknowledgments Section of your manuscript:

“This work was supported by National Institutes of Health (NIH) grant 1R21CA259985 (LW, LS, SRH, HLH, AET, CJP, DGS), P30CA016058 (DGS, LW, TS, AET), 1R01 CA215151 (AET), CJP is supported by 1K76AG074923-01.”

We note that you have provided funding information that is not currently declared in your Funding Statement. However, funding information should not appear in the Acknowledgments section or other areas of your manuscript. We will only publish funding information present in the Funding Statement section of the online submission form.

Please remove any funding-related text from the manuscript and let us know how you would like to update your Funding Statement. Currently, your Funding Statement reads as follows:

“This work was supported by National Institutes of Health (NIH) grant 1R21CA259985 (LW, LS, SRH, HLH, AET, CJP, DGS); HealthyState Alliance Momentum Grant (DGS); National Institutes of Health (NIH) grant P30CA016058 (DGS, LW, TS, AET), National Institutes of Health (NIH) grant 1R01 CA215151 (AET) National Institutes of Health (NIH) grant to CJP is supported by 1K76AG074923-01. The sponsors or funders play any role in the study design, data collection and analysis, decision to publish, or preparation of the manuscript. http://www.cancer.gov”

Please include your amended statements within your cover letter; we will change the online submission form on your behalf.

4. Thank you for stating the following in the Competing Interests section:

“HH is on the scientific advisory board for Invitae Genetics, Genome Medical, and Promega. She has stock/stock options in Genome Medical and GI OnDemand. LS is a consultant and speaker for AstraZeneca. DS served on an advisory board for Novartis, Guardant, and receives research funding (in kind to institution) from Foundation Medicine and NeoGenomics. CJP received payment for patient education material development through Jazz Pharmaceuticals.”

We note that you received funding from a commercial sources:

• AstraZeneca

• Novartis

• Guardant

• NeoGenomics

• Jazz Pharmaceuticals

Please provide an amended Competing Interests Statement that explicitly states this commercial funder, along with any other relevant declarations relating to employment, consultancy, patents, products in development, marketed products, etc.

Within this Competing Interests Statement, please confirm that this does not alter your adherence to all PLOS ONE policies on sharing data and materials by including the following statement: "This does not alter our adherence to PLOS ONE policies on sharing data and materials.” (as detailed online in our guide for authors http://journals.plos.org/plosone/s/competing-interests). If there are restrictions on sharing of data and/or materials, please state these. Please note that we cannot proceed with consideration of your article until this information has been declared.

Please include your amended Competing Interests Statement within your cover letter. We will change the online submission form on your behalf.

5. Please provide a complete Data Availability Statement in the submission form, ensuring you include all necessary access information or a reason for why you are unable to make your data freely accessible. If your research concerns only data provided within your submission, please write "All data are in the manuscript and/or supporting information files" as your Data Availability Statement.

6. Your ethics statement should only appear in the Methods section of your manuscript. If your ethics statement is written in any section besides the Methods, please move it to the Methods section and delete it from any other section. Please ensure that your ethics statement is included in your manuscript, as the ethics statement entered into the online submission form will not be published alongside your manuscript.

7. Please upload a new copy of Figures 1 a to 1d; and Supplementary Figure 1 as the detail is not clear. Please follow the link for more information: https://journals.plos.org/plosone/s/figures

8. Please include captions for your Supporting Information files at the end of your manuscript, and update any in-text citations to match accordingly. Please see our Supporting Information guidelines for more information: http://journals.plos.org/plosone/s/supporting-information.

If the reviewer comments include a recommendation to cite specific previously published works, please review and evaluate these publications to determine whether they are relevant and should be cited. There is no requirement to cite these works unless the editor has indicated otherwise.

Additional Editor Comments (if provided):

The authors attempted to respond to previous comments yet the original concerns persist.

The authors were not sufficiently responsive to reviewers.

Please provide scientifically proofing and convincing point-by-point responses to the reviewers and revise the manuscript accordingly.

[Note: HTML markup is below. Please do not edit.]

Reviewers' comments:

Reviewer's Responses to Questions

**Comments to the Author**

Reviewer #1: (No Response)

Reviewer #2: All comments have been addressed

Reviewer #3: All comments have been addressed

Reviewer #4: All comments have been addressed

**********

2. Is the manuscript technically sound, and do the data support the conclusions?

Reviewer #1: Partly

Reviewer #2: Yes

Reviewer #3: (No Response)

Reviewer #4: Yes

**********

3. Has the statistical analysis been performed appropriately and rigorously?

Reviewer #1: Yes

Reviewer #2: Yes

Reviewer #3: (No Response)

Reviewer #4: Yes

**********

4. Have the authors made all data underlying the findings in their manuscript fully available?

The PLOS Data policy

Reviewer #1: Yes

Reviewer #2: Yes

Reviewer #3: (No Response)

Reviewer #4: Yes

**********

5. Is the manuscript presented in an intelligible fashion and written in standard English?

Reviewer #1: Yes

Reviewer #2: Yes

Reviewer #3: (No Response)

Reviewer #4: Yes

**********

Reviewer #1: The authors attempted to respond to previous comments yet the original concerns persist as follows:

1. It does not appear that the video was viewed and evaluated by patients/caregivers before deployment. While developed with patient input, having the same or additional patients evaluate the video would increase confidence that the video met the information needs it intended to meet.

2. The VMSK does not appear to have been validated. The authors do a good job explaining where the items originated, there is no description of cognitive testing (in which people not involved in survey development respond in detail to what they think they are being asked). This is how one confirms that the instrument is measuring what it is designed to measure. It does not appear that the instrument has been validated. Validation requires several steps, including comparing responses to other published instruments to see if responses are concordant with (at least in some aspect) with previously-used and validated instruments.

3. The authors concur that attending to the needs of people with lower levels of education is an important area to address. There is nothing in the revised manuscript that attends to this concern other than reiterating the intention of including community practices. This reviewer would encourage the authors to not only expand their sample but also to consult with and incorporate the views and comprehension of this important target population through iterative work with their patient community.

Reviewer #2: Authors have adequately responded to queries of all three reviewers and so i see no barriers to acceptance.

Reviewer #3: (No Response)

Reviewer #4: Dear Authors,

This a well-executed manuscript evaluating the impact of a brief educational intervention designed to improve patient understanding of tumor genomic testing (TGT). The main findings demonstrate that this video significantly improved patient knowledge related to the specific educational messages conveyed in the video, which were maintained through the return of TGT results. The study addresses an important gap by assessing both the immediate and longer-term effects of a concise educational video across multiple cancer types and practice settings.

Notably, while video message-specific knowledge improved significantly, objective genomic knowledge and trust in providers remained unchanged, suggesting that the intervention was effective in communicating key practical concepts but did not have an effect on broader genomic literacy or patient-provider trust.

Overall, this study provides important evidence supporting the use of brief, standardized educational materials to enhance patient understanding of TGT and may serve as a model for improving implementation of genomic medicine in diverse clinical settings.

Overall, this manuscript in its current iteration has addressed all of the important issues raised by the previous reviews. Therefore, I have minor edits/suggestions at this time:

1. Line 88: The field is moving away from using the terminology "mutation", the authors should consider alternatives such as "targeted therapies guided by genomic alterations", "genomically guided therapies" or "molecularly targeted therapies"

2. Lines 115-116: Please rephrase for clarity, "…,some specifically do not want testing". Do the authors mean that patients wants to be notified but do not want to pursue confirmatory germline testing? Or some want to be notified of secondary findings but some do not?

3. In relation to reviewer 1, comment 3 can the authors add some detail on how they determined that the TGT was discussed during the visit in order to administer T3? As we know, TGT results are not always discussed and if the TGT was uninformative, they may have spend follow-up appointments on other items.

4. Do the authors have any insight into why those who had highly penetrant suspected germline findings were not referred?

5. Line 300-301: The option to opt-out of germline results is not always available and will depend on TGT vendor and state regulations. Please change to "reviewing potential options to opt-out"

Thank you for inviting me to review this interesting study.

**********

what does this mean?). If published, this will include your full peer review and any attached files.

If you choose “no”, your identity will remain anonymous but your review may still be made public.

**Do you want your identity to be public for this peer review?**  For information about this choice, including consent withdrawal, please see our Privacy Policy

Reviewer #1: No

Reviewer #2: No

Reviewer #3: No

Reviewer #4: **Yes:**  Ilana B. Solomon

**********

[NOTE: If reviewer comments were submitted as an attachment file, they will be attached to this email and accessible via the submission site. Please log into your account, locate the manuscript record, and check for the action link "View Attachments". If this link does not appear, there are no attachment files.]

To ensure your figures meet our technical requirements, please review our figure guidelines: https://journals.plos.org/plosone/s/figures

You may also use PLOS’s free figure tool, NAAS, to help you prepare publication quality figures: https://journals.plos.org/plosone/s/figures#loc-tools-for-figure-preparation.

NAAS will assess whether your figures meet our technical requirements by comparing each figure against our figure specifications.

# Author comment: Author response to Decision Letter 2

**DOI:** 10.1371/journal.pone.0360611.r005

15 Sep 2026

Application of a Concise Video to Improve Patient Understanding of Tumor Genomic Testing in Community and Academic Practice Settings

Deloris J. Veney MACPR1, Lai Wei, PhD, MS2; Jaden Miller3; Amanda E. Toland, Ph D3,4; Carolyn J. Presley, MD, MHS1; Heather Hampel, MS5; Tasleem J. Padamsee, Ph D6; Michael J. Bishop, MD7; James J Kim, MD8; Shelly R. Hovick, Ph D9; William J. Irvin, Jr., MD10; Leigha Senter, MS3+; Daniel G. Stover, MD1,2,11+

POINT-BY-POINT RESPONSE TO REVIEWS

Editorial Comments

1. Please ensure that your manuscript meets PLOS ONE's style requirements, including those for file naming.

Confirmed

2. We note that the grant information you provided in the ‘Funding Information’ and ‘Financial Disclosure’ sections do not match.

When you resubmit, please ensure that you provide the correct grant numbers for the awards you received for your study in the ‘Funding Information’ section.

Thank you – we have reconciled this (below)

3. Thank you for stating the following in the Acknowledgments Section of your manuscript:

“This work was supported by National Institutes of Health (NIH) grant 1R21CA259985 (LW, LS, SRH, HLH, AET, CJP, DGS), P30CA016058 (DGS, LW, TS, AET), 1R01 CA215151 (AET), CJP is supported by 1K76AG074923-01.”

We note that you have provided funding information that is not currently declared in your Funding Statement. However, funding information should not appear in the Acknowledgments section or other areas of your manuscript. We will only publish funding information present in the Funding Statement section of the online submission form.

Please remove any funding-related text from the manuscript and let us know how you would like to update your Funding Statement. Currently, your Funding Statement reads as follows:

“This work was supported by National Institutes of Health (NIH) grant 1R21CA259985 (LW, LS, SRH, HLH, AET, CJP, DGS); HealthyState Alliance Momentum Grant (DGS); National Institutes of Health (NIH) grant P30CA016058 (DGS, LW, TS, AET), National Institutes of Health (NIH) grant 1R01 CA215151 (AET) National Institutes of Health (NIH) grant to CJP is supported by 1K76AG074923-01. The sponsors or funders play any role in the study design, data collection and analysis, decision to publish, or preparation of the manuscript. http://www.cancer.gov”

Please include your amended statements within your cover letter; we will change the online submission form on your behalf.

Thank you. We have removed it from the Acknowledgements and reconciled. The amended Funding Statement is:

“This work was supported by National Institutes of Health (NIH) grant 1R21CA259985 (LW, LS, SRH, HLH, AET, CJP, DGS); HealthyState Alliance Momentum Grant (DGS); National Institutes of Health (NIH) grant P30CA016058 (DGS, LW, TS, AET); National Institutes of Health (NIH) grant 1R01CA215151 (AET); National Institutes of Health (NIH) grant 1K76AG074923 (CJP).

The sponsors or funders did not play any role in the study design, data collection and analysis, decision to publish, or preparation of the manuscript.”

4. Thank you for stating the following in the Competing Interests section:

“HH is on the scientific advisory board for Invitae Genetics, Genome Medical, and Promega. She has stock/stock options in Genome Medical and GI OnDemand. LS is a consultant and speaker for AstraZeneca. DS served on an advisory board for Novartis, Guardant, and receives research funding (in kind to institution) from Foundation Medicine and NeoGenomics. CJP received payment for patient education material development through Jazz Pharmaceuticals. ”

We note that you received funding from a commercial sources:

• AstraZeneca

• Novartis

• Guardant

• NeoGenomics

• Jazz Pharmaceuticals

Please provide an amended Competing Interests Statement that explicitly states this commercial funder, along with any other relevant declarations relating to employment, consultancy, patents, products in development, marketed products, etc.

Within this Competing Interests Statement, please confirm that this does not alter your adherence to all PLOS ONE policies on sharing data and materials by including the following statement: "This does not alter our adherence to PLOS ONE policies on sharing data and materials.” (as detailed online in our guide for authors http://journals.plos.org/plosone/s/competing-interests). If there are restrictions on sharing of data and/or materials, please state these. Please note that we cannot proceed with consideration of your article until this information has been declared.

Please include your amended Competing Interests Statement within your cover letter. We will change the online submission form on your behalf.

The amended Competing Interests Statement now reads:

“HH is on the scientific advisory board for Invitae Genetics, Genome Medical, and Promega. She has stock/stock options in Genome Medical and GI OnDemand. LS is a consultant and speaker for AstraZeneca. DS served on an advisory board for Novartis, Guardant, and receives research funding (in kind to institution) from Foundation Medicine and NeoGenomics. CJP received payment for patient education material development through Jazz Pharmaceuticals. This does not alter our adherence to PLOS ONE policies on sharing data and materials.”

5. Please provide a complete Data Availability Statement in the submission form, ensuring you include all necessary access information or a reason for why you are unable to make your data freely accessible. If your research concerns only data provided within your submission, please write "All data are in the manuscript and/or supporting information files" as your Data Availability Statement.

Data Availability Statement updated in the submission form.

6. Your ethics statement should only appear in the Methods section of your manuscript. If your ethics statement is written in any section besides the Methods, please move it to the Methods section and delete it from any other section. Please ensure that your ethics statement is included in your manuscript, as the ethics statement entered into the online submission form will not be published alongside your manuscript.

This now only appears in the Methods.

7. Please upload a new copy of Figures 1 a to 1d; and Supplementary Figure 1 as the detail is not clear. Please follow the link for more information: https://journals.plos.org/plosone/s/figures

New copies of Figure 1 and Supplementary Figure 1 have been updated/uploaded

8. Please include captions for your Supporting Information files at the end of your manuscript, and update any in-text citations to match accordingly. Please see our Supporting Information guidelines for more information: http://journals.plos.org/plosone/s/supporting-information.

If the reviewer comments include a recommendation to cite specific previously published works, please review and evaluate these publications to determine whether they are relevant and should be cited. There is no requirement to cite these works unless the editor has indicated otherwise.

Noted.

Additional Editor Comments (if provided):

The authors attempted to respond to previous comments yet the original concerns persist.

The authors were not sufficiently responsive to reviewers.

Please provide scientifically proofing and convincing point-by-point responses to the reviewers and revise the manuscript accordingly.

Thank you – we believe that we have convincingly responded as below. Specifically, Reviewers 2-4 indicated “All comments have been addressed” and we responded in detail to Reviewer #1’s remaining critiques.

Reviewers' comments:

Reviewer's Responses to Questions

Comments to the Author

1. If the authors have adequately addressed your comments raised in a previous round of review and you feel that this manuscript is now acceptable for publication, you may indicate that here to bypass the “Comments to the Author” section, enter your conflict of interest statement in the “Confidential to Editor” section, and submit your "Accept" recommendation.

Reviewer #1: (No Response)

Reviewer #2: All comments have been addressed

Reviewer #3: All comments have been addressed

Reviewer #4: All comments have been addressed

2. Is the manuscript technically sound, and do the data support the conclusions?

The manuscript must describe a technically sound piece of scientific research with data that supports the conclusions. Experiments must have been conducted rigorously, with appropriate controls, replication, and sample sizes. The conclusions must be drawn appropriately based on the data presented.

Reviewer #1: Partly

Reviewer #2: Yes

Reviewer #3: (No Response)

Reviewer #4: Yes

3. Has the statistical analysis been performed appropriately and rigorously?

Reviewer #1: Yes

Reviewer #2: Yes

Reviewer #3: (No Response)

Reviewer #4: Yes

4. Have the authors made all data underlying the findings in their manuscript fully available?

The PLOS Data policy requires authors to make all data underlying the findings described in their manuscript fully available without restriction, with rare exception (please refer to the Data Availability Statement in the manuscript PDF file). The data should be provided as part of the manuscript or its supporting information, or deposited to a public repository. For example, in addition to summary statistics, the data points behind means, medians and variance measures should be available. If there are restrictions on publicly sharing data—e.g. participant privacy or use of data from a third party—those must be specified.

Reviewer #1: Yes

Reviewer #2: Yes

Reviewer #3: (No Response)

Reviewer #4: Yes

5. Is the manuscript presented in an intelligible fashion and written in standard English?

PLOS ONE does not copyedit accepted manuscripts, so the language in submitted articles must be clear, correct, and unambiguous. Any typographical or grammatical errors should be corrected at revision, so please note any specific errors here.

Reviewer #1: Yes

Reviewer #2: Yes

Reviewer #3: (No Response)

Reviewer #4: Yes

6. Review Comments to the Author

Please use the space provided to explain your answers to the questions above. You may also include additional comments for the author, including concerns about dual publication, research ethics, or publication ethics. (Please upload your review as an attachment if it exceeds 20,000 characters)

Reviewer #1: The authors attempted to respond to previous comments yet the original concerns persist as follows:

1.. While developed with patient input, It does not appear that the video was viewed and evaluated by patients/caregivers before deployment having the same or additional patients evaluate the video would increase confidence that the video met the information needs it intended to meet.

Thank you to the Reviewer for commenting on this point.

This video has NOT yet been deployed outside of a research setting.

The goal of this study is to have additional patients evaluate the video (in a research design) to confirm that the video meets the information needs it indents to meet. Thus, the study population of 203 participants viewing the video where we establish the video as an evidence-based intervention prior to clinical use.

In addition, the video was viewed by cancer care providers for various cancer types, presented at Grand Rounds at OSU and BSMH, and to nursing staff at all institutions. It was created as a collaboration between Genetic Counseling, Medical Oncology, and with the input of an adult health and risk communication expert.

We did not hold a second round of focus groups. Our original focus groups and interviewees were metastatic cancer patients. Due to the amount of time that it took to create the video and the reality of stage IV cancer it was not feasible to return to the same patients for additional input.

In the preliminary results, respondents confirmed that the video met the information needs:

“A Likert scale of agreement was used to measure participant's opinions of the video's utility, clarity, and engagement (Figure S4C), and most (>80%) of respondents agreed or strongly agreed that the video was helpful, easy to understand, and held their attention, with fewer but still most (60%) indicating that the graphics were helpful. Most participants (141 of 150) felt that the amount of information presented was adequate, while four participants felt the content was excessive, and five participants found the content insufficient.”

-From: Veney DJ, Wei LY, Toland AE, et al. A video intervention to improve patient understanding of tumor genomic testing in patients with cancer. Cancer Med. 2024; 13:e70095. doi:10.1002/cam4.70095

2. The VMSK does not appear to have been validated. The authors do a good job explaining where the items originated, there is no description of cognitive testing (in which people not involved in survey development respond in detail to what they think they are being asked). This is how one confirms that the instrument is measuring what it is designed to measure. It does not appear that the instrument has been validated. Validation requires several steps, including comparing responses to other published instruments to see if responses are concordant with (at least in some aspect) with previously-used and validated instruments.

We agree with the reviewer regarding the importance of using validated instruments and employed validated instruments whenever possible, including GKU, TIPP, and knowledge and knowledge insufficiency from the Risk Information Seeking and Processing Model.

There was no previously validated measure to directly assess knowledge change obtained through our video intervention. The initial development of the True/False VMSK items, taken directly from the video script, were grounded in preliminary quality improvement work, including patient focus groups, qualitative interviews, and provider surveys, to ensure content validity and clear understanding of the core concepts prior to research instrument deployment at OSU. This is published work: Senter L, Veney D, Surplus T, et al. Patient Understanding of Tumor Genomic Testing: A Quality Improvement Effort. JCO Oncol Pract. 2023;19(1):e8-e14. doi:10.1200/OP.22.00316.

We agree with the reviewer that comparison to validated instruments is critical and, as part of the study design, the VMSK and the validated GKU instruments were administered simultaneously to all participants at T1 and T2. This dual-question design was intended to allow us to demonstrate concordance at T1, and by repeating both GKU and VMSK at T2, also allow us to attribute pre-test/post-test score change in VMSK solely to the video Intervention.

While participants demonstrated a statistically significant increase in VMSK scores following the educational intervention (median increase of 10 points; p<0.0001), their scores on the published GKU survey remained completely unchanged pre- to post-video (p=0.89). This demonstrated both convergent validity, the VMSK sensitive to message-specific knowledge gain, and construct validity, the intervention specifically improved knowledge related to the video content rather than triggering generalized genomic literacy changes.

“As an internal control, genomic knowledge/understanding did not significantly change suggesting that knowledge gained was specifically related to the video.”

We have now revised the Discussion section of the manuscript submission to read.

“The absence of a previously-validated survey instrument that directly addresses tumor genomic testing is a limitation to this study because individual question response concordance could not be directly assessed. Knowledge concordance between VMSK and GKU provided a surrogate measure for VMSK instrument validation purposes. VMSK provides insight into the key material specifically included in the video, while GKU serves as a previously validated baseline measure and concurrent benchmark (alongside the VMSK items) to evaluate participant performance. GKU provides an alternative set of related data that were not specifically included in the video, thus serving as the validation control metric for the VMSK instrument.”

In summary, by using this combination of previously validated instruments in conjunction with our VMSK instrument, we simultaneously use the validated instruments to gather data on the video intervention and provide validity for the VMSK survey.

3. The authors concur that attending to the needs of people with lower levels of education is an important area to address. There is nothing in the revised manuscript that attends to this concern other than reiterating the intention of including community practices. This reviewer would encourage the authors to not only expand their sample but also to consult with and incorporate the views and comprehension of this important target population through iterative work with their patient community.

Thank you to the reviewer for this explanation of their concern. As noted, in Table 1, we demonstrate that through the addition of the Community cohort, we added to the demographic divisions that had lower levels of education: those categories that represent individuals that did not graduate high school and those whose formal education terminated at high school graduation. High school graduates are the majority of this cohort (versus our other three cohorts where the majority of respondents claimed education beyond high school).

We also comment within the manuscript “Interestingly, while the T1:T2 absolute improvement was the greatest in Cohort 1: Breast, the T1:T3 absolute improvement was greatest in Cohort 4: Community (Fig 1A), suggesting that different groups (for example, those with distinct educational attainment) may benefit and retain the information differently.”

The addition of the Community cohort, a central element of this study, represents the iterative work that the reviewer suggests. We certainly saw that across all 203 participants, those with lowest education attainment demonstrated lowest T1 VMSK scores and shallowest knowledge gains, approximately 7 pts vs 9 pts for all other brackets. We presented this in the Results section:

Association of Baseline Patient Characteristics with Survey Outcomes

The association of patient characteristics with baseline VMSK, GKU, and TIPP were assessed via Spearman correlations (Table 2). Across the entire study population there was a significant association between education and baseline/T1 VMSK (nominal p<0.0001) and GKU (nom p=0.004) as well as income and baseline/T1 GKU (nom p=0.007) and GKU (nom p=0.004).

Because we saw knowledge gains across all educational divisions, we were confident that despite the T1/T2 12-pt+ score separations between participants with least-to-most formal education, all cohorts would receive benefit from video viewing. The score changes were consistent with our previous analysis so we focused on the new information from the community cohort: their knowledge recall was the most improved. The following statement was added to the Results section “Mean VMSK score gains across the educational demographic divisions of some high school education or less, high school graduate or equivalent, some higher education, and college graduate were 7.14, 9.05, 7.09, and 11.08 respectively.” And “With all education demographic divisions showing VMSK score improvement, we demonstrate that the video intervention provides an educational resource for learners of varied educational attainment and does not negatively impact comprehension surrounding TGT for any bracket.” was added to the Discussion section.

(Reviewer #2: Authors have adequately responded to queries of all three reviewers and so i see no barriers to acceptance.

Reviewer #3: (No Response)

Reviewer #4:

Dear Authors,

This a well-executed manuscript evaluating the impact of a brief educational intervention designed to improve patient understanding of tumor genomic testing (TGT). The main findings demonstrate that this video significantly improved patient knowledge related to the specific educational messages conveyed in the video, which were maintained through the return of TGT results. The study addresses an important gap by assessing both the immediate and longer-term effects of a concise educational video across multiple cancer types and practice settings.

Notably, while video message-specific knowledge improved significantly, objective genomic knowledge and trust in providers remained unchanged, suggesting that the intervention was effective in communicating key practical concepts but did not have an effect on broader genomic literacy or patient-provider trust.

Overall, this study provides important evidence supporting the use of brief, standardized educational materials to enhance patient understanding of TGT and may serve as a model for improving implementation of genomic medicine in diverse clinical settings.

Overall, this manuscript in its current iteration has addressed all of the important issues raised by the previous reviews. Therefore, I have minor edits/suggestions at this time:

1. Line 88: The field is moving away from using the terminology "mutation", the authors should consider alternatives such as "targeted therapies guided by genomic alterations", "genomically guided therapies" or "molecularly targeted therapies"

Thank you to the reviewer for this insight and for the rephrasing suggestions. These changes have been incorporated from lines 87-95.

2. Lines 115-116: Please rephrase for clarity, "…,some specifically do not want testing". Do the authors mean that patients wants to be notified but do not want to pursue confirmatory germline testing? Or some want to be notified of secondary findings but some do not?

Thank you to the Reviewer for pointing out where clarity was needed. In fact, the three publications cited in regard to this statement reflect that some patients simply do not want any genomic testing while others do not want follow-up testing for incidental findings. The reasons for this remain unclear and it was our hope that with education, patients would see the value of testing and avoid confusion with genetic testing. This sentence now reads “some patients do not want initial genomic testing and/or follow-up genetic testing if incidental findings are discovered.”

3. In relation to reviewer 1, comment 3 can the authors add some detail on how they determined that the TGT was discussed during the visit in order to administer T3? As we know, TGT results are not always discussed and if the TGT was uninformative, they may have spend follow-up appointments on other items.

Thank you to the reviewer for this question. An early obstacle to study performance was the lack of provider follow-up of TGT results. It is one element that affects the duration of time between T1/T2 -T3. The following addition has been made to the Methods section of the manuscript “Study personnel were embedded in clinic teams to remind providers to discuss the results of TGT with patients and to verify that those discussions took place prior to participants being requested to complete the T3 survey. Participants were only offered the T3 survey if chart documentation was present that indicated that TGT results were communicated to the patient or if the Provider verbally confirmed with the survey administrator that the requisite conversations took place.”

4. Do the authors have any insight into why those who had highly penetrant suspected germline findings were not referred?

Thank you to the Reviewer for posing this important question. This was investigated separately at OSU through quality improvement data gathering where the rate of omission was similar (abstract in preparation), suggesting that this phenomenon was not limited to the current study population. Efforts to improve that specific metric are being handled within that same QI endeavor. We hypothesize that this may be a combination of factors, including provider knowledge, patient awareness, and time in clinic – among other potential factors. In the absence of firm data, we noted, “Our prior work suggests that incidental germline findings are not discussed routinely in oncology care largely due to time constraints.“

5. Line 300-301: The option to opt-out of germline results is not always available and will depend on TGT vendor and state regulations. Please change to "reviewing potential options to opt-out"

Thank you to the Reviewer for volunteering this information and corrective phrasing. This suggestion has been incorporated in the manuscript. Line 300-301, now line 323-324, reads “These organizations echo the importance of informed consent prior to TGT and reviewing potential options for patients to opt out of receipt of germline results.”

Thank you for inviting me to review this interesting study.

Attachment Submitted filename: Response to Reviewers_Final.docx

# Peer review: Decision Letter 2

**DOI:** 10.1371/journal.pone.0360611.r006

**Authors:** Kokouvi Kassegne

29 Sep 2026

Application of a Concise Video to Improve Patient Understanding of Tumor Genomic Testing in Community and Academic Practice Settings

PONE-D-26-13280R2

Dear Dr. Daniel Stover,

We’re pleased to inform you that your manuscript has been judged scientifically suitable for publication and will be formally accepted for publication once it meets all outstanding technical requirements.

Within one week, you’ll receive an e-mail detailing the required amendments. When these have been addressed, you’ll receive a formal acceptance letter and your manuscript will be scheduled for publication.

An invoice will be generated when your article is formally accepted. Please note, if your institution has a publishing partnership with PLOS and your article meets the relevant criteria, all or part of your publication costs will be covered. Please make sure your user information is up-to-date by logging into Editorial Manager at Editorial Manager® and clicking the ‘Update My Information' link at the top of the page. For questions related to billing, please contact billing support.

If your institution or institutions have a press office, please notify them about your upcoming paper to help maximize its impact. If they’ll be preparing press materials, please inform our press team as soon as possible -- no later than 48 hours after receiving the formal acceptance. Your manuscript will remain under strict press embargo until 2 pm Eastern Time on the date of publication. For more information, please contact onepress@plos.org.

Kind regards,

Kokouvi Kassegne

Academic Editor

PLOS One

Additional Editor Comments (optional):

The authors have been responsive and have clarified the limitations of the work; I therefore recommend the manuscript for publication

Reviewers' comments:

Reviewer's Responses to Questions

**Comments to the Author**

Reviewer #1: All comments have been addressed

**********

2. Is the manuscript technically sound, and do the data support the conclusions?

Reviewer #1: Yes

**********

3. Has the statistical analysis been performed appropriately and rigorously?

Reviewer #1: Yes

**********

4. Have the authors made all data underlying the findings in their manuscript fully available?

The PLOS Data policy

Reviewer #1: Yes

**********

5. Is the manuscript presented in an intelligible fashion and written in standard English?

Reviewer #1: Yes

**********

Reviewer #1: (No Response)

**********

what does this mean?). If published, this will include your full peer review and any attached files.

If you choose “no”, your identity will remain anonymous but your review may still be made public.

**Do you want your identity to be public for this peer review?**  For information about this choice, including consent withdrawal, please see our Privacy Policy

Reviewer #1: No

**********

# Editor report: Acceptance letter

**DOI:** 10.1371/journal.pone.0360611.r007

**Authors:** Kokouvi Kassegne

PONE-D-26-13280R2

PLOS One

Dear Dr. Stover,

I'm pleased to inform you that your manuscript has been deemed suitable for publication in PLOS One. Congratulations! Your manuscript is now being handed over to our production team.

At this stage, our production department will prepare your paper for publication. This includes ensuring the following:

* All references, tables, and figures are properly cited

* All relevant supporting information is included in the manuscript submission,

* There are no issues that prevent the paper from being properly typeset

You will receive further instructions from the production team, including instructions on how to review your proof when it is ready. Please keep in mind that we are working through a large volume of accepted articles, so please give us a few days to review your paper and let you know the next and final steps.

Lastly, if your institution or institutions have a press office, please let them know about your upcoming paper now to help maximize its impact. If they'll be preparing press materials, please inform our press team within the next 48 hours. Your manuscript will remain under strict press embargo until 2 pm Eastern Time on the date of publication. For more information, please contact onepress@plos.org.

You will receive an invoice from PLOS for your publication fee after your manuscript has reached the completed accept phase. If you receive an email requesting payment before acceptance or for any other service, this may be a phishing scheme. Learn how to identify phishing emails and protect your accounts at https://explore.plos.org/phishing.

If we can help with anything else, please email us at customercare@plos.org.

Thank you for submitting your work to PLOS One and supporting open access.

Kind regards,

PLOS One Editorial Office Staff

on behalf of

Dr. Kokouvi Kassegne

Academic Editor

PLOS One
